# Verification of our empirical understanding of the physiology and ecology of two contrasting plantation species using a trait database

**DOI:** 10.1371/journal.pone.0254599

**Published:** 2021-11-29

**Authors:** Yoko Osone, Shoji Hashimoto, Tanaka Kenzo

**Affiliations:** 1 Forestry and Forest Products Research Institute, Tsukuba, Japan; 2 Japan International Research Center for Agricultural Sciences, Tsukuba, Japan; Universidade Federal de Viçosa, BRAZIL

## Abstract

The effects of climate change on forest ecosystems take on increasing importance more than ever. Information on plant traits is a powerful predictor of ecosystem dynamics and functioning. We reviewed the major ecological traits, such as foliar gas exchange and nutrients, xylem morphology and drought tolerance, of *Cryptomeria japonica* and *Chamaecyparis obtusa*, which are major timber species in East Asia, especially in Japan, by using a recently developed functional trait database for both species (SugiHinokiDB). Empirically, *C*. *obtusa* has been planted under drier conditions, whereas *C*. *japonica*, which grows faster but thought to be less drought tolerant, has been planted under wetter conditions. Our analysis generally support the empirical knowledge: The maximum photosynthetic rate, stomatal conductance, foliar nutrient content and soil-to-foliage hydraulic conductance were higher in *C*. *japonica* than in *C*. *obtusa*. In contrast, the foliar turgor loss point and xylem pressure corresponding to 50% conductivity, which indicate drought tolerance, were lower in *C*. *obtusa* and are consistent with the drier habitat of *C*. *obtusa*. Ontogenetic shifts were also observed; as the age and height of the trees increased, foliar nutrient concentrations, foliar minimum midday water potential and specific leaf area decreased in *C*. *japonica*, suggesting that nutrient and water limitation occurs with the growth. In *C*. *obtusa*, the ontogenetic shits of these foliar traits were less pronounced. Among the Cupressaceae worldwide, the drought tolerance of *C*. *obtusa*, as well as *C*. *japonica*, was not as high. This may be related to the fact that the Japanese archipelago has historically not been subjected to strong dryness. The maximum photosynthetic rate showed intermediate values within the family, indicating that *C*. *japonica* and *C*. *obtusa* exhibit relatively high growth rates in the Cupressaceae family, and this is thought to be the reason why they have been selected as economically suitable timber species in Japanese forestry. This study clearly demonstrated that the plant trait database provides us a promising opportunity to verify out empirical knowledge of plantation management and helps us to understand effect of climate change on plantation forests by using trait-based modelling.

## 1. Introduction

There is an emerging scientific consensus that the global climate change is resulting in decreased stability in forest ecosystems [1, 2]. The effects of climate change on the forestry sector have been examined in general terms for many regions of the world but rarely with sufficient temporal or spatial resolution to influence regional or local forest management [3–8]. The major issues include how the ranges in which commercially important tree species are suitable for plantations will change in the future and whether climatic influence can be overridden by appropriate forest management. Answering these questions requires a basic understanding of the physiology and ecology of target tree species.

Information on plant traits, that is, any physiological, morphological or phenological features measurable at the individual level [9], is now widely used to predict how forests will respond to future climate change [10–13]. Process-based models generally use leaf-scale process, such as, photosynthetic capacity, stomatal response to vapor pressure deficit (VPD) and respiration of a plant species or a functional type for modelling C dynamics under given climate scenarios [8, 14]. There are also studies focusing on traits more directly related to drought sensitivity for predicting future hydraulic risk. For example, leaf water potential at turgor loss (Ψtlp), which had been recognized a classical index of plant water stress, was demonstrated to be a powerful indicator of drought tolerance within and across biomes [15, 16], while hydraulic safety margins, defined as difference between minimum xylem water potential and water potential at which 50% loss of conductivity occurs (Ψ50) is becoming widely used for a predictor of drought-induced tree mortality [17, 18]. Clearly, trait information holds promise for better understanding of the vulnerability to drought, as well as parameterizing models with increased robustness and accuracy.

With the increasing demands for trait information, trait databases are becoming key research tools in this study field [13]. One of the strengths of trait databases is that they provide a wide array of traits for a species all at once, which is generally difficult in a single study since measurements of physiological and morphological properties are time- and labour-intensive. Another advantage is that they show the variability within a species since they store data from different studies which measured plants at different ages in different locations. Many functional traits change ontogenetically as plants grow [19–25]. Understanding the ontogenetic drift of key functional traits is important for impact assessments of climate change since forest management is a long-term commitment and requires optimality of adaptation strategies at each growth stage [26, 27].

Recently, we created a trait database for Japanese cedar (*Cryptomeria japonica* D. Don, *Cupressaceae*) and Japanese cypress (*Chamaecyparis obtusa* (Siebold et Zucc.) Endl., *Cupressaceae*) (SugiHinokiDB), which contains 24683 data for 177 plant traits compiled from diverse sources, such as papers, bulletins, reports and books [28]. *C*. *japonica* and *C*. *obtusa* produce high-quality wood and have been the most important commercial tree species in Japan. They were also introduced for timber production in many regions of the world: China, Korean Peninsula, India, Nepal, Azores and Réunion [29, 30]. In Japan, these species were planted to forest sites according to empirically derived rule for species selection. *C*. *japonica*, which grows faster but thought to be less drought tolerant than *C*. *obtusa*, is traditionally planted on moist and nutrient-rich sites, whereas *C*. *obtusa* is planted on relatively dry and nutrient-poor sites [31–33]. Since these management practices had worked well until recently, we have paid little attention on the physiological mechanisms underlying their habitat preferences. However, without the knowledge, we cannot predict how the species respond to climate change, nor what the optimal adaptation strategies are. SugiHinokiDB, which compiled traits that are closely related to the life history strategy [13, 34–37], with a special focus on traits related to water relations, may offer a comprehensive characterization of the growth and hydraulics of these species.

In this study, using the SugiHinoki DB, we verify the empirical knowledge that *C*. *japonica* grows faster but is less tolerant to drought than *C*. *obtusa* based on three steps:

1. We selected 20 traits that are central to the leading dimensions of plant strategy and quantified the differences in those traits between the two species. Our hypothesis is that *C*. *japonica* has relatively pioneer-like properties, i.e., a higher gas exchange rate, specific leaf area (m^2^ g^−1^, SLA), and xylem and foliar water conductivity, whereas *C*. *obtusa* shows more conservative resource use and a higher drought tolerance.
2. We also examined the ontogenetic changes in some foliage traits. There is still limited knowledge on age or height depending changes in foliage traits, particularly their species patterns. We demonstrated how leaf traits are changed with ontogeny (age or height) between different interspecific degrees of drought tolerance.
3. Finally, we compare some hydraulic properties of these species with those of Cupressaceae worldwide. Cupressaceae species are thought to differentiate along an aridity gradient and vary greatly in their drought sensitivity [38]. We discussed the adaptive strategies of *C*. *japonica* and *C*. *obtusa* in light of the phylogenetic lineage and potential as timber species under future climate.

## 2. Materials and methods

### 2.1 Plant species

Japanese cedar (*Cryptomeria japonica* (L.f.) D. Don, Sugi cedar), an evergreen conifer, is the only species of the genus *Cryptomeria* in Cupressaceae; it is distributed mainly in Japan. In Zhejiang, China, a small population of closely related species of *C*. *fortune* is distributed; however, these populations may have been introduced from Japan for timber species, and genetic analysis supported this assumption [39]. In Japan, its natural range is Lat. 30°to 40°N with mean annual precipitation > 1800 mm [40]. *C*. *japonica*, which grows rapidly with a maximum height greater than 50 m, has been used for timber production since the prehistoric period. At present, it dominates approximately 45% of the forest area in Japan. Japanese cypress (*Chamaecyparis obtusa* (Sieb. et Zucc.) Endl.), distributed in Japan and Taiwan, is also an evergreen conifer in the *Cupressaceae* family. The northern limit of its natural range (Lat. 30°-37°N) is lower in latitude than that of *C*. *japonica*. Due to low snow resistance, the species rarely appear coastal area of Sea of Japan, where there is plenty of snow in winter. Although *C*. *obtusa* grows slower than *C*. *japonica*, it produces high-quality wood and thus has also long been a commercially important species in Japan. *Chamaecyparis obtusa* dominates 15% of the forest area in Japan.

### 2.2 Plant trait database

The sugi-hinoki database (SugiHinoki DB) consists of 24683 data entries for 177 traits of *C*. *japonica* and *C*. *obtusa* from 364 primary sources such as journal papers, bulletins, reports and books including unpublished data and grey literature [28]. Since most data are obtained from published material, they go through initial quality assurance [41, 42]. The unpublished data and grey literature were checked for their Materials and Methods, and if there was no doubt about the measurement method or data, they were adopted into the database. The traits, grouped into 15 categories by their features (Table 1), are those that are widely agreed on as relevant to plant life-history strategies, vegetation modelling and global change responses [34, 36, 37]. Because of the limited distribution of the species, data were mainly obtained from forest sites in Japan but also from arboretums or plantations in Taiwan, Korea and China. The database includes data from plants grown in plantation forests, natural forests, and those grown under experimental conditions. Each data entry is accompanied by ancillary information about the location, environmental conditions, experimental treatment, measurement methods, status of measured individuals in the stand and the position of measured parts (e.g., upper or lower crown for photosynthetic measurements). Further details on the database are given in [28].

**Table 1:** List of 108 major plant traits selected from the SugiHinoki DB.

			*Cryptomeria japonica*			*Chamaecyparis obtusa*		
Category	Trait	Unit	n	Mean	Range	n	Mean	Range
Photosynthesis	Maximum photosynthesis per foliage area (Amaxan, Amaxas)	μmol m^-2^ s^-1^	216	6.7	0.3–19.9	215	5.3	0.4–11.3
	Maximum photosynthesis rate per foliage dry mass	μmol kg^-1^ s^-1^	844	30.9	0.7–120.5	141	29.5	0.2–84.5
	Maximum rate of electron transport per foliage area (Jmaxa)	μmol m^-2^ s^-1^	66	135.4	46.0–260.6	18	76.4	49.4–95.6
	Maximum rate of electron transport per foliage dry mass	μmol kg^-1^ s^-1^	62	447.7	119.9–911.9	0	—	
	Maximum rate of carboxylation per foliage area (Vcmaxa)	μmol m^-2^ s^-1^	228	59.2	20.3–139.1	34	31.1	14.7–51.1
	Maximum rate of carboxylation per foliage dry mass	μmol kg^-1^ s^-1^	62	228.9	50.4–493.6	0	—	
	Initial slope of light response curve	molCO_2_ mol[e]^-1^	48	0.030	0.016–0.074	33	0.050	0.020–0.-81
	Convexity of light response curve	NA	45	0.60	0.04–0.98	30	0.66	0.09–0.93
	Light compensation point of photosynthesis	μmol[e] m^-2^ s^-1^	7	53.1	32.4–87.5	17	26.3	11.0–37.7
	Mass content of chlorophyll per foliage dry mass	mg g^-1^	243	2.68	0.66–7.48	0	—	
	Ratio of chlorophyll a to chlorophyll b.	NA	45	2.43	1.59–3.69	42	2.61	2.34–2.99
	Mass content of Rubisco per foliage dry mass	mg g^-1^	3	19.5	12.4–26.6	0	—	
Respiration	Leaf dark respiration rate per area	μmol m^-2^ s^-1^	128	0.76	0.07–2.76	223	0.65	0.02–3.10
	Leaf dark respiration rate per foliage dry mass	μmol kg^-1^ s^-1^	155	4.40	0.44–24.00	3	12.82	5.46–17.0
	Day respiration rate per foliage area	μmol m^-2^ s^-1^	39	5.45	1.50–14.27	0	—	
	Branch dark respiration rate per branch volume	μmol m^-3^ s^-1^	46	378	56–2051	26	274	21–1405
	Stem dark respiration rate per stem surface area	μmol m^-2^ s^-1^	207	3.67	0.22–12.46	89	1.55	0.24–4.44
	Root dark respiration rate per dry mass	μmol kg^-1^ s^-1^	26	4.57	2.52–7.32	23	6.10	2.80–9.75
	Fineroot dark respiration rate per dry mass	μmol kg^-1^ s^-1^	0	—		107	8.51	3.80–16.0
	Q10 measured for foliage	NA	0	—		30	2.43	1.87–3.65
	Q10 measured for stem	NA	18	1.93	1.45–2.81	0	—	
Water relation	Stomatal conductance for CO2 per foliage area (gsan)	mol m^-2^ s^-1^	59	0.10	0.01–0.93	40	0.08	0.001–0.52
	Stomatal conductance for CO2 per foliage dry mass	mol kg^-1^ s^-1^	139	0.43	0.03–2.00	24	0.43	0.22–0.65
	Transpiration rate per foliage area (Ean)	mmol m^-2^ s^-1^	76	1.08	0.03–3.30	95	2.77	0.11–17.0
	Transpiration rate per foliage dry mass	mmol kg^-1^ s^-1^	395	12.1	0.10–102.1	207	6.19	0.01–41.7
	Sap flow density/velocity measured by Granier method.	cm^3^ m^-2^ s^-1^	88	17.3	0.48–40.6	19	17.9	6.1–46.1
	Sap frow density averaged for a day	cm^3^ m^-2^ s^-1^	29	8.4	3.0–20.0	24	11.4	4.0–19.4
	Sap flow (or transpiration) per a tree per a day	kg d^-1^	112	9.4	0.7–22.9	176	12.6	1.1–67.2
	Soil to leaf hydraulic conductance per foliage area (KS-L)	mmol m^-2^ s^-1^ Mpa^-1^	37	0.6	0.2–1.3	9	1.1	0.3–1.7
	Soil to leaf hydlauric resistance per foliage mass	Mpa kg s mmol^-1^	64	1.2	0.1–5.8	60	1.2	0.0–10.1
	Stem (sapwood) specific conductivity (Kstem)	kg m^-1^ s^-1^ MPa^-1^	18	1.0	0.4–2.2	9	1.3	0.3–2.3
	Predawn foliage water potential (Ψpd)	MPa	51	-0.45	-2.14- -0.02	132	-0.63	-2.28- -0.01
	Midday foliage water potential (Ψmd)	MPa	370	-1.03	-2.30- -0.05	189	-1.29	-2.49- -0.28
	Osmotic potential at water saturation (Ψπsat)	MPa	258	-1.60	-2.70- -0.70	93	-1.27	-2.38- -0.11
	Leaf water potential at turgor loss point (Ψtlp)	MPa	262	-2.31	-3.55- -1.31	161	-2.33	-4.09- -0.91
	Water content at turgor loss point (RWCtlp)	g g^-1^	101	0.76	0.65–0.86	93	0.68	0.53–0.81
	Bulk elastic modulus (ℇ)	MPa	101	7.4	1.7–15.1	53	4.4	0.6–10.7
	Branch water potential at 50% conductivity loss (Ψ50)	MPa	2	-4.8	-5.2- -4.4	2	-6.7	-7.6- -5.8
	Root water potential at 50% conductivity loss	MPa	1	-4.1	-4.1- -4.1	2	-3.1	-4.3- -2.0
	Water use efficiency	mg g^-1^	8	4.9	3.6–5.4	8	5.2	4.2–6.6
	13C:12C ratio in leaves	‰	74	-27.4	-30.1- -24.7	44	-26.8	-28.5- -24.6
Leaf morphology	Specific leafe area (SLA)	m^2^ kg^-1^	379	4.9	1.5–16.9	244	5.8	3.2–15.7
	Shoot silhouette area to projected needle area ratio (SPAR)	NA	39	0.6	0.5–0.7	0	—	
Root morphology	Specific root length	m g^-1^	132	13.3	0.2–48.4	196	14.8	0.3–46.2
	Specific root surface area	cm^2^ mg^-1^	44	0.16	0.03–0.37	3	0.19	0.03–0.43
Anatomy	Number of stomata per foliage area	mm^-2^	173	127.9	11.9–19.9	7	635.3	500–830
	Width of stomata	μm	3	65.8	31.2–342.0	2	20.7	19.9–21.5
	Length of stomata	μm	21	47.9	28.0–85.3	2	28.2	27.4–29.0
	Cross sectional area of foliage xylem	μm^2^	43	1639	1048–111.9	33	434	213–1104
	Cross sectional area of foliage transfusion cell	μm^2^	46	9369	3522–16917	34	367	43–1082
	Tracheid length of early wood	mm	8	2.4	1.3–3.0	99	2.3	0.9–3.6
	Tracheid length of late wood	mm	495	2.4	0.8–4.5	7	2.8	1.8–3.8
	Tracheid diameter (tangent) of early wood	μm	47	28.2	21.6–37.0	3	24.0	21.0–26.0
	Tracheid diameter (radial) of early wood	μm	56	35.2	24.9–51.6	3	27.0	24.0–29.0
	Tracheid diameter (tangent) of late wood	μm	32	25.5	19.8–30.1	3	19.0	16.0–22.0
	Tracheid diameter (radial) of late wood	μm	56	15.0	9.0–24.9	3	14.7	13.0–16.0
Wood density	Basic density of stem	g cm^3^	640	0.35	0.13–0.59	44	0.41	0.38–0.50
	Basic density of branch	g cm^3^	31	0.32	0.06–0.65	0	—	
	Basic density of root	g cm^3^	4	0.42	0.39–0.45	4	0.46	0.40–0.50
	Basic density of fine root	g cm^3^	44	0.28	0.17–0.33	105	0.29	0.17–0.80
Resource use	Nitrogen resorption efficiency of leaves	%	3	23.1	5.5–49.1	19	39.4	23.4–56.4
	N content of foliage litter fall	mg g^-1^	6	7.8	6.4–8.3	52	7.8	4.3–11.3
	15N:14N ratio in leaves	‰	4	2.0	-4.9–9.0	4	2.3	-6.4–10.3
	Leaf longevity	year	24	4.7	2.9–10.4	47	4.6	1.9–8.3
	Fineroot longevity	year	1	4.1	4.1–4.1	7	2.3	1.3–5.3
Nitrogen content	Nitrogen content per foliage area (Na, Ns)	g m^-2^	381	3.91	0.97–12.6	112	2.54	0.77–4.11
	Nitrogen content per foliage dry mass (Nm)	mg g^-1^	2116	13.8	4.50–44.3	759	14.1	6.74–32.4
	Nitrogen content per stem dry mass	mg g^-1^	163	5.03	0.30–16.2	32	8.08	0.40–15.5
	Nitrogen content per root dry mass	mg g^-1^	152	7.84	0.50–27.5	41	11.9	4.90–18.6
	Nitrogen content per thick root (>20mm) dry mass	mg g^-1^	28	1.64	0.30–3.20	4	1.23	0.20–2.40
	Nitrogen content per thin root (<20mm, >2mm) dry mass	mg g^-1^	28	3.40	0.60–5.10	4	3.60	3.00–4.30
	Nitrogen content per fine root (<2mm) dry mass	mg g^-1^	63	9.78	2.90–18.2	107	10.4	0.92–17.0
Phosphorous content	Phosphorus content per foliage dry mass (Pm)	mg g^-1^	692	1.52	0.51–8.90	357	1.58	0.40–8.10
	Phosphorus content per stem dry mass	mg g^-1^	163	0.51	0.04–3.52	32	0.47	0.08–1.10
	Phosphorus content per root dry mass	mg g^-1^	145	0.85	0.13–7.28	34	1.22	0.57–2.79
	Phosphorus content of thick root (>20mm) dry mass	mg g^-1^	28	0.32	0.13–0.70	4	0.39	0.17–0.65
	Phosphorus content of thin root (<20mm, >2mm) dry mass	mg g^-1^	28	0.37	0.17–0.74	4	0.26	0.17–0.49
	Phosphorus content of fine root (<2mm) dry mass	mg g^-1^	26	0.55	0.26–1.35	4	0.60	0.31–0.79
Pottasium content	Pottasium content per foliage mass (Km)	mg g^-1^	722	8.10	1.77–37.2	354	7.96	2.31–36.4
	Pottasium content per stem dry mass	mg g^-1^	163	3.05	0.17–12.2	32	4.15	0.58–7.05
	Pottasium content per root dry mass	mg g^-1^	157	3.71	0.17–16.8	34	4.09	1.41–14.8
	Pottasium content per thick root (>20mm) dry mass	mg g^-1^	28	1.22	0.66–2.49	4	1.02	0.83–1.16
	Pottasium content per thin root (<20mm, >2mm) dry mass	mg g^-1^	28	1.87	1.08–2.57	4	1.49	1.41–1.74
	Pottasium content per fine root (<2mm) dry mass	mg g^-1^	26	2.20	1.08–4.31	4	2.05	1.33–2.82
Chemical composition	Lignin content of foliage	%	3	25.9	23.6–28.3	4	24.4	23.6–25.6
	Lignin content of foliage litter fall	%	2	34.8	30.2–39.4	1	27.3	27.3–27.3
	Lignin content of deadwood	%	69	33.5	30.2–40.6	67	31.3	28.4–38.1
	Holocellulose content of foliage	%	3	48.1	47.4–48.9	4	51.6	50.7–53.9
	Holocellulose content of foliage litter fall	%	2	46.5	45.5–47.5	1	42.4	42.4–42.4
	Holocellulose content of deadwood	%	69	63.5	55.0–68.1	67	65.6	57.7–69.0
	Nitrogen content of deadwood	g kg^-1^	69	0.87	0.22–4.46	67	0.55	0.10–2.12
	Carbon content of deadwood	g kg^-1^	69	514	483–537	67	522	498–547
Biomass	Leaf biomass (LM)	kg	150	4.1	0.001–57.1	77	4.82	0.090–22.2
	Stem+branch biomass	kg	150	30.3	0.00003–477.2	77	43.3	1.65–208.4
	Branch biomass	kg	72	6.15	0.0003–39.0	77	4.79	0.111–23.2
	Root biomass (RM)	kg	195	7.15	0.00004–146.4	100	9.48	0.00002–67.8
	Thick root biomass (>20mm)	kg	29	25.3	0.83–138.9	16	15.9	2.18–59.5
	Thin root biomass (<20mm, >2mm)	kg	29	3.26	0.46–6.00	16	3.47	1.15–6.79
	Fine root biomass (<2mm)	kg	39	0.46	0.0008–1.54	16	0.68	0.20–1.47
Allocation	Annual allocation of net primary production to foliage	t ha^-1^ y^-1^	94	4.78	1.70–7.70	22	3.77	2.80–5.35
	Annual allocation of net primary production to stem + branch	t ha^-1^ y^-1^	94	9.60	2.10–28.6	22	9.85	6.40–13.6
	Annual allocation of net primary production to branch	t ha^-1^ y^-1^	80	1.82	0.36–5.10	21	2.03	0.60–2.80
	Annual allocation of net primary production to root	t ha^-1^ y^-1^	94	2.68	0.53–7.38	22	2.84	1.70–3.90
	Annual foliage litterfall	t ha^-1^ y^-1^	46	3.61	0.93–6.64	130	3.16	0.83–5.96
	Annual branch + bark litterfall	t ha^-1^ y^-1^	26	0.83	0.09–3.70	83	0.69	0.02–7.04
Stand characteristics	Leaf area index (LAI)	NA	16	0.20	2.07–17.2	62	7.00	2.03–12.4
	LAI-based light absorption coefficient (k)	NA	6	0.85	0.26–0.46	1	0.97	0.97–0.97
	Leaf mass-based Light absorption coefficient	NA	2	0.26	0.25–0.27	2	0.25	0.24–0.27

Data number, means and ranges were calculated before outliers were removed for the following analyses.

Table 1 lists 108 major plant traits selected from the SugiHinoki DB. The traits with the highest number of data entries are mass-based nutrient contents such as mass-based foliar N concentration (N_m,_ 2875), K concentration (K_m_, 1076) and P concentration (P_m_, 1049), which had once been easy-to-measure indices of plant physiological status. Other basic foliar properties, including photosynthetic capacities (Amax_a_, 431; Amax_m_, 985), SLA (623), foliar pressure-volume curve parameters (Ψ_tlp_, 423; RWC_tlp_ 194; Ψ_πsat_, 351; ℇ, 154) and midday (minimum) foliage water potential (Ψ_md_, 559), are also those with the highest number of data entries. On the other hand, fewer data are available for properties that require more complex measurements, such as water conductance/conductivity and embolism resistance. *C*. *japonica* and *C*. *obtusa* showed similar patterns in data abundance among traits, but most traits of *C*. *japonica* had more data than *C*. *obtusa* (81 out of 108 traits). Each trait of a species showed quite large variation in the values since the database contains data for trees of different ages, grown under different conditions and measured at different times of the day or year. The data distribution of each trait showed a convex curve when data were abundant, but distributions were mostly positively skewed.

### 2.3 Selection of traits for the comparison of *C*. *japonica* and *C*. *obtusa*

We selected 20 traits from SugiHinokiDB to detect the ecophysiological basis for the empirical knowledge that *C*. *japonica* grows faster on nutrient-rich moist soil than *C*. *obtusa*. The selected traits are those that reflect ecological characteristics and adaptability to the environment [43] and that had enough sample size to identify species differences, except for several traits on drought tolerance. Those include the maximum photosynthetic rate per area (Amax_a_), maximum carboxylation rate per area (Vcmax_a_), maximum electron transport rate per area (Jmax_a_), foliar dark respiration rate per area (R_a_), stomatal conductance for CO_2_ per area (gs_a_), foliar N per area (N_a_), SLA, foliar water potential at the turgor loss point (Ψ_tlp_), foliar relative water content at the turgor loss point (RWC_tlp_), foliar osmotic potential at full turgor (Ψ_πsat_), bulk elastic modulus (ε), soil-to-foliage water conductance (K_S-L_), stem specific conductivity (K_stem_), tracheid diameter of the stem, tracheid length of the stem, basic density of the stem, xylem water potential at a 50% loss of conductivity (Ψ_50_), foliage mass (LM), stem mass (SM) and root mass (RM). Abbreviations and units of the traits used in the analyses are shown in Table 1. SugiHinokiDB also reserves information on relationships between traits shown in original sources. For some traits, especially those related to water use, we investigate the trait interaction to gain more insight in their adaptive strategy [44].

### 2.4 Selection of traits for age or height dependency

For the analysis of age or height dependency, we used the foliage nitrogen content per foliage dry mass (N_m)_, foliage phosphorus content per foliage dry mass (P_m_), foliage potassium content per foliage dry mass (K_m_), specific leaf area (SLA) and midday foliage water potential (Ψ_md_). These are key traits for plant growth and water relations, and changes in these traits with age or size could have considerable effects on stand growth, carbon and nutrient cycling, and thus forest management. In SugiHinoki DB, they are abundant in data entries from many sources where measurements were performed for many trees of different ages or sizes. However, since the data were not obtained from a single carefully controlled experiment but were the compilation of multiple studies, we could not separate size and age effects that may independently affect foliage traits.

### 2.5 Two measures of foliage-area based traits

**Fig 1:**
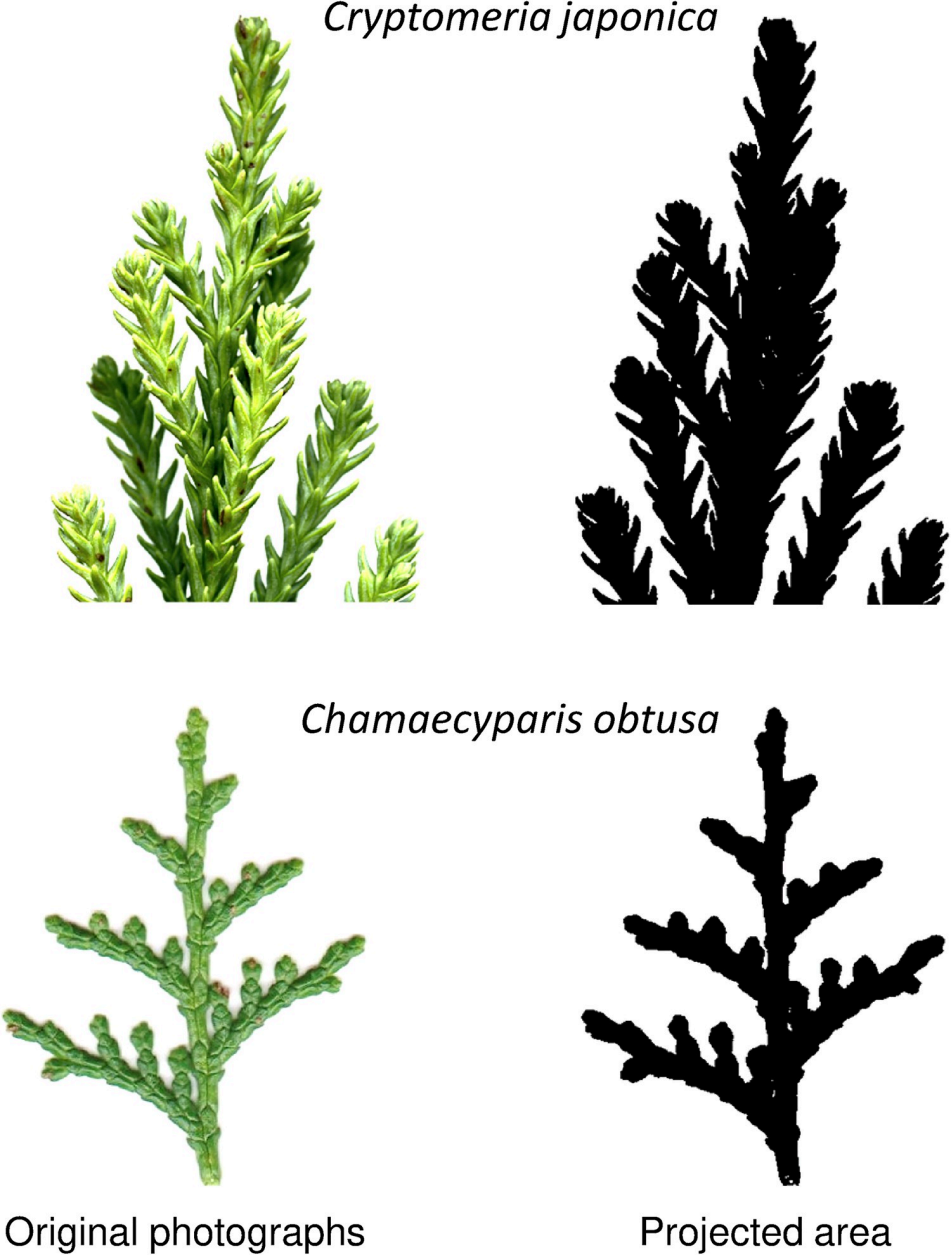
Photographs of *Cryptomeria japonica* (top) and *Chamaecyparis obtusa* (bottom). The right side is an example of the foliar projected area. Note that the projected area is underestimated in *C*. *japonica* because the foliage of *C*. *japonica* has a complex three-dimensional structure.

Shoots of *C*. *japonica* have complex structures, with needles being attached densely and helicoidally to a stalk, while shoots of *C*. *obtusa* are planar with scale-like leaves arranged on a flat surface (Fig 1). As a result, in *C*. *japonica*, there are large differences in projected needle area and the shoot silhouette area, and thus the area-based foliar traits differ largely depending on whether the ‘foliage area’ is projected needle (or scale) area (A_n_) or the shoot silhouette area (A_s_). The relationship between the trait values presented on a needle area basis (T_leaf_/A_n_) and shoot silhouette area basis (T_leaf_/A_s_) for any trait (T_leaf_) is given as

$$\frac{T_{leaf}}{A_{n}}=SPAR\frac{T_{leaf}}{A_{s}},$$ (1)

where SPAR is the shoot silhouette and projected needle area ratio [45, 46] and is given as

$$SPAR=\frac{A_{s}}{A_{n}},$$ (2)

The SPAR is less than 1 in conifers with complex shoot structures and is 1 in broad-leaf species and conifers with planar shoots, such as *C*. *obtusa*. A smaller SPAR means higher needle clumping or higher mutual shading in a shoot. SPAR can vary among species and the crown position of a tree [46–48]. However, [49] found that the relationships between needle area and shoot projected area were almost consistent among shoots of different ages and different sizes among 4 cultivars of *C*. *japonica*, with a slope (= SPAR) of 0.63.

Similarly, the relationship between needle area-based SLA (An/M_n_) and shoot silhouette area-based SLA (As/M_s_) is

$$\frac{A_{n}}{M_{n}}=\frac{A_{s}}{M_{s}}\frac{1}{SPAR}\frac{M_{s}}{M_{n}}$$ (3)

where M_n_ and M_s_ are the needle mass of a shoot and the mass of a shoot, respectively. In SugiHinokiDB, these two values were stored individually. In this analysis, we focused on both needle/scale area-based (presented with suffix ‘n’ after trait abbreviation such as ‘Amax_an_’) and shoot silhouette area-based traits (presented with suffix ‘s’ after trait abbreviation such as ‘Amax_as_’) since the two could have different ecological meanings, especially in terms of light capture [45, 47, 48, 50, 51].

### 2.6 Data filtering

For all selected data, errors were checked, and outliers, which we defined as data out of accepted ranges, were excluded. An accepted range was calculated by the method of interquartile range (IQR) for log-transformed data as [36, 52]:

Q1 − 1.5× IQR < accepted range < Q3 + 1.5 × IQR

where Q1 is the first and Q3 is the third quartile of the data, and IQR is given by:

IQR = Q3-Q1.

The number of data excluded as outliers accounts for 3.4% of the whole data of the database. We then excluded data collected under experimental conditions (plants grown in a greenhouse, growth chamber and lysimeter; plants grown in planters; plantation forests with experimental fertilization, etc.), which resulted in the exclusion of most young plants (0–2 years). For ecophysiological traits (e.g. photosynthesis, stomatal conductance, transpiration, and midday water potential), which fluctuate largely with internal and external factors, we selected data measured under the condition with which maximum rates are achieved. In other words, we used data obtained from current and one-year-old foliage with sun-lit during growing season (Jun-Oct). Afternoon data were also excluded to avoid the effect of the midday depression on gas exchange by stomatal limitation, except for midday water potential.

### 2.7 Statistical analysis

Differences between species were tested by Student’s t-test. For foliage traits, where we used two measures (needle- or shoot-base) for *C*. *japonica* and one measure (scale-base) for *C*. *obtusa*, differences were tested between species, i.e., each of the two measures for *C*. *japonica* was compared with the scale-traits for *C*. *obtusa*. The relationship between organ biomass and DBH/total biomass were assessed by regression analysis and the differences in regressions between species were tested by analysis of covariance. The age and height dependency of the foliar traits were also assessed by regression analysis. Species difference in the seasonal changes in PV parameters were tested by ANOVA. All statistical analyses were performed with R Version 3.4.4 [53].

## 3. Results and discussion

### 3.1 Differences in traits related to growth rate

#### 3.1.1 Photosynthesis

**Fig 2:**
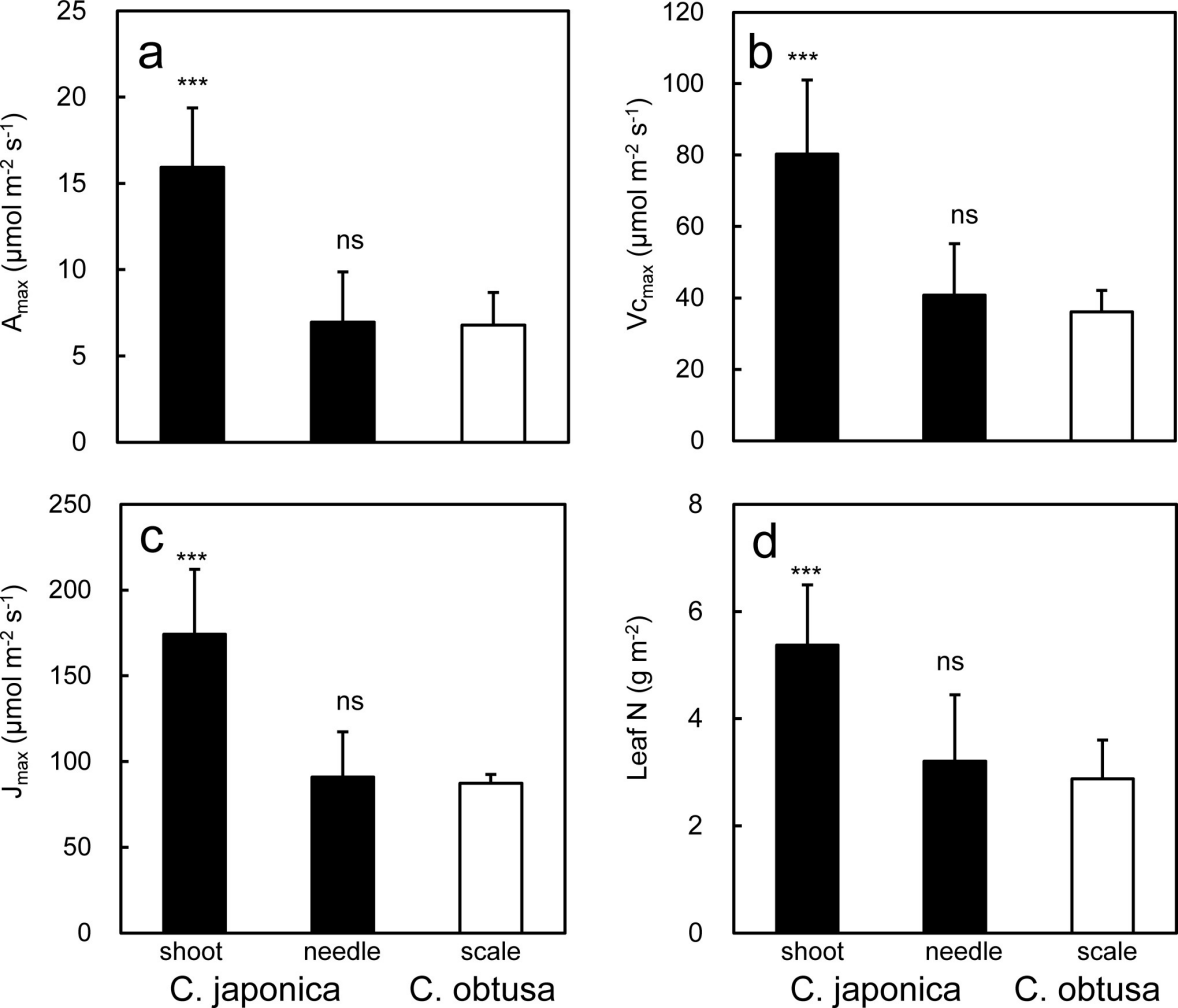
Photosynthetic properties of *Cryptomeria japonica* and *Chamaecyparis obtusa*. In *C*. *japonica*, each trait is presented on a shoot silhouette area basis and projected needle area basis. Each shoot or needle-based trait for *C*. *japonica* was compared with the scale traits for *C*. *obtusa* by *t*-test; ***, *P* < 0.001; ns, not significant.

In contrast to our hypothesis that photosynthetic capacity and foliar (needle or scale) N concentration are higher in fast-growing *C*. *japonica* than in slow-growing *C*. *obtusa*, they were not significantly different between the species when presented on a foliage area basis (Amax_an_, Vcmax_an_, Jmax_an_, N_an_) (Fig 2, compare “needle” of *C*. *japonica* and “scale” of *C*. *obtusa*). However, since the two species have completely different shoot morphologies (Fig 1), a simple comparison of these foliage area-based measurements may fail to characterize their photosynthetic properties [54]. Photosynthetic capacity is usually presented per unit foliage area on the assumption that the foliage area represents the amount of solar radiation intercepted. However, the interception of solar radiation is not directly related to the total foliage area unless foliage is oriented horizontally. In *C*. *japonica*, where needles are attached helicoidally to a stalk, mutual shading could occur within the shoots, which reduces the photosynthetic rate per needle area [45, 48, 50]. In such shoots, as in many other coniferous shoots, light interception is determined by the shoot silhouette area rather than the total needle area, and photosynthetic characteristics should be evaluated based on both photosynthesis per needle area and per shoot silhouette area [50].

On a shoot silhouette basis, the photosynthetic capacity (Amax_as_, Vcmax_as_, Jmax_as_) and foliar N contents (N_as_) were higher for *C*. *japonica* than for *C*. *obtusa* (Amax_an_, Vcmax_an_, Jmax_an_, N_an_), by 1.9–2.1 times (Fig 2, compare “shoot” for *C*. *japonica* and “scale” for *C*. *obtusa*). The marked increases in the photosynthetic capacity of *C*. *japonica* when presented based on the shoot silhouette area are explained by Eq 1. Amax_as_ is Amax_an_, divided by SPAR (Eq 1). Since SPAR is 0.53–0.73 in *C*. *japonica* (SugiHinoki DB), Amax_as_ could be 1.4–1.9 times higher than Amax_an._ Note that the two measures of photosynthesis are consistent in *C*. *obtusa* in which shoot is planar and SPAR is one. The higher Amax_as_ in *C*. *japonica* than in *C*. *obtusa* despite the similar Amax_an_ suggests that under saturating irradiance, densely packed needles on the shoots of *C*. *japonica* can absorb more irradiance than the planar shoots of *C*. *obtusa* and thereby achieve a higher photosynthetic rate per shoot. However, this could be at the expense of the photosynthetic efficiency of each needle in *C*. *japonica*; that is, at low irradiance, the photosynthetic rate per needle area could be decreased more than that of *C*. *obtusa* due to mutual shading of needles.

This is analogous to the effects that the different anatomy of sun/shade leaves has on foliar photosynthesis or the different structure of grass stands (steep foliage angle)/forb type (vertical foliage) on canopy photosynthesis [55, 56]. Similar to sun leaves with thick tissue layers and grass stands with high LAI, needle clumping would be favourable only if incident light is high and penetrates deep into the shoots. Under these conditions, whole shoot productivity could be higher than that of planar shoots where shoot photosynthesis saturates at lower light levels. If the incident light is low, however, light attenuates on the upper layers of the shoots without being transmitted to deeper layers. Under these circumstances, planar shoots are preferable for higher efficiency of weak light capture just as shade leaves or forb-type stands are preferred under low light availability. The differences in the light interception between the two shoot types were also reflected in photosynthetic light response curves. *C*. *japonica* had a lower initial slope (0.031) and convexity (0.59) than *C*. *obtusa* (initial slope, 0.048; convexity, 0.66) since photosynthesis increases and saturates at a slower rate with increasing irradiance in three-dimensional shoots (data from SugiHinokiDB). This is a well-documented pattern in the photosynthetic light response curves of sun/shade leaves and indicates that *C*. *obtusa* has characteristics of shade leaves in comparison with *C*. *japonica* [43]. Although our hypothesis that photosynthetic capacity would be higher in *C*. *japonica* than in *C*. *obtusa* was not supported on a per needle basis, their adaptation to different light environments is more apparent in their light use and photosynthesis at the shoot level.

The two shoot types may also differ in total light interception per day. Light interception is the most efficient when leaves are oriented to face the direction of the light source [43]. Therefore, planar shoots (*C*. *obtusa*) can intercept light more efficiently than shoots that have needles with various orientations (*C*. *japonica*) when the light source is just above them and is weak. However, since the solar azimuth angle changes considerably during a day, shoots that have needles with various orientations may be able to intercept more light and have higher assimilation rate on a daily basis than planar shoots, especially under strong light conditions [45, 50]. These results also imply that *C*. *japonica* is advantageous over *C*. *obtusa* in open habitats where strong light is available for longer times during a day. Such environments do appear in early stages of plantations before canopy closure. Young *C*. *japonica* saplings showed almost twice higher diameter growth rate than *C*. *obtusa* under open conditions, whereas only a 10% higher growth rate in *C*. *japonica* was observed under darker conditions, such as <3% of relative light intensity [57]. Thus, *C*. *japonica* may be able to grow faster than *C*. *obtusa* due to higher daily photosynthesis in this stage of a plantation.

#### 3.1.2 SLA

**Fig 3:**
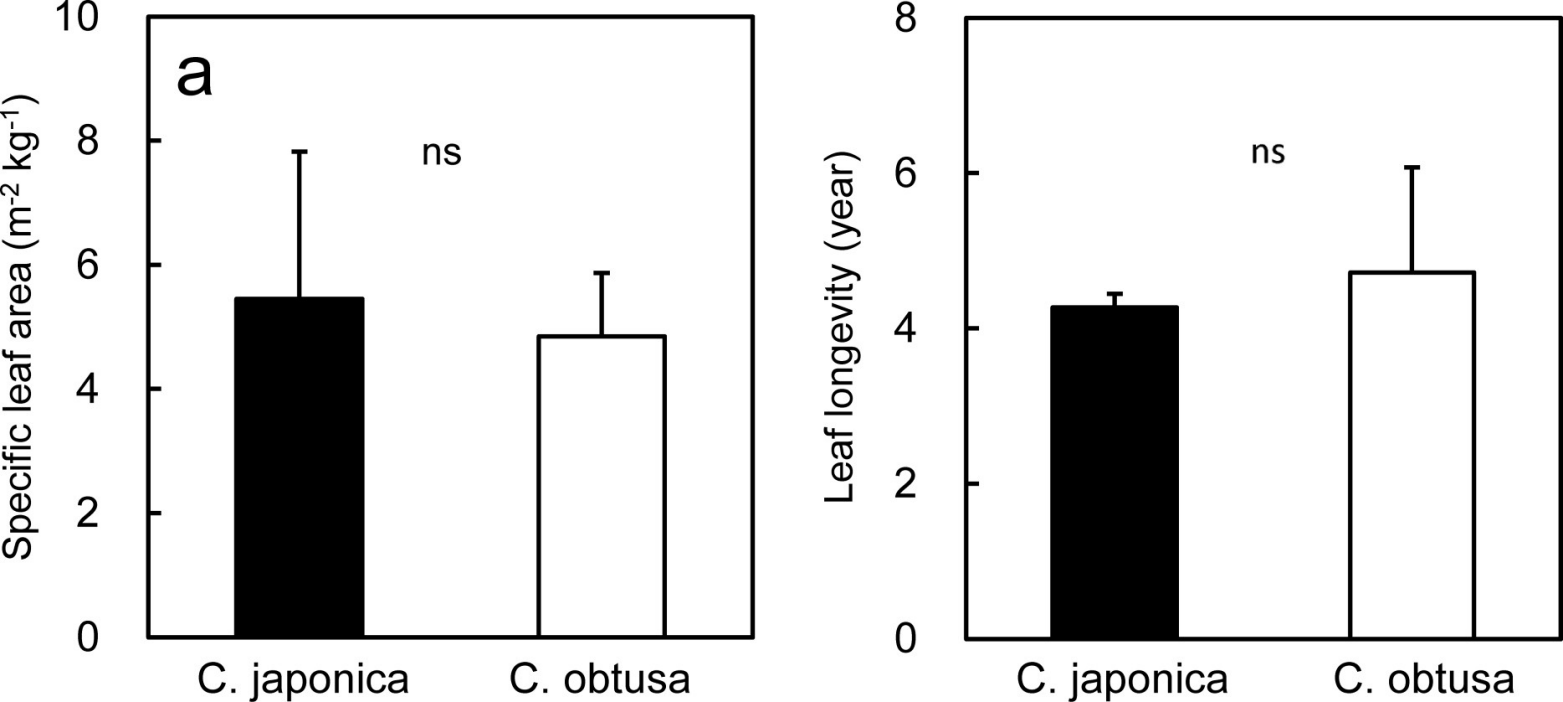
Specific leaf area and leaf longevity of *Cryptomeria japonica* and *Chamaecyparis obtusa*. Differences between *C*. *japonica* and *C*. *obtusa* were tested by a t-test; ns, not significant.

SLA (projected foliage area per foliage mass) was not significantly different between *C*. *japonica* and *C*. *obtusa* (Fig 3A). This contradicts our hypothesis and the vast majority of studies that have reported a correlation between SLA and the relative growth rate across a wide range of plant species [58–64]. From the viewpoint of growth analysis, SLA contributes to the relative growth rate because a high SLA is assumed to represent a large photosynthetic surface (= area of light interception) per given foliage biomass [43, 65]. However, as discussed above, the relationship between needle area and light interception is not straightforward for three-dimensional shoots, and it is possible that *C*. *japonica*, with shoots of various needle orientations, has a higher daily assimilation rate than *C*. *obtusa* with planar shoots in certain environments. On the other hand, the consistent SLA of the two species appears reasonable from the viewpoint of the leaf economic spectrum, a multivariable correlation between key chemical, structural and physiological properties of leaves based on the carbon and nitrogen economy (e.g., [66]). One of the key axes of the leaf economic spectrum is that SLA is positively correlated with the mass based photosynthetic capacity and foliage N concentration and is negatively correlated with foliage longevity [67]. If so, the two species with a similar photosynthetic capacity (Fig 2A–2C, needle basis), foliage N concentration (Fig 2D, needle basis) and foliage longevity (Fig 3B), should also be similar in SLA.

#### 3.1.3 Biomass ratio between foliage and other organs

**Fig 4:**
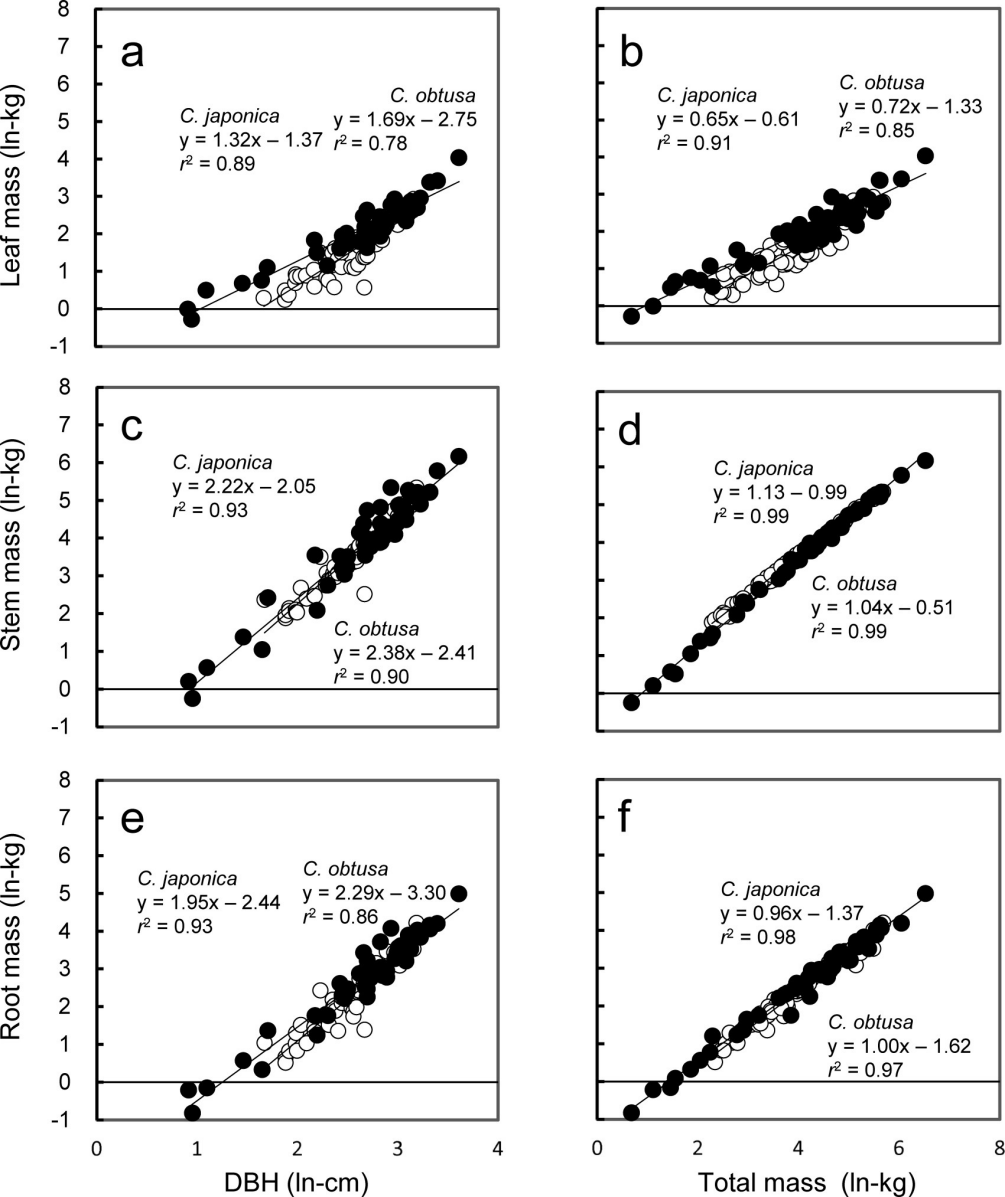
Organ biomass in relation to DBH (a, c, e) and total mass (b, d, f). Differences in regressions between species were tested by analysis of covariance (ANCOVA, [69]). There were significant interspecific differences between leaf biomass and DBH or total biomass (*P* < 0.0001) and stem biomass and total biomass (*P* < 0.05), though the other relations were similar between species (P > 0.05).

The biomass ratio between organs is also a factor that affects the growth rate because a relatively high foliar mass equates to a relatively large photosynthetic organ if other factors are equal [43, 65]. At a given DBH or total biomass, the stem and root biomasses were not significantly different between the species (*P* > 0.05, ANCOVA, Fig 4), except a slight difference in intercept was detected between stem and total biomass (*P* < 0.05, ANCOVA, Fig 4). In contrast, foliar biomass was significantly higher in *C*. *japonica* than in *C*. *obtusa* (*P* < 0.0001, ANCOVA, Fig 4). This tendency was more pronounced at smaller DBHs or total biomass, suggesting that the initial greater foliar mass ratio (LMR) could contribute to faster growth at early stages of growth in *C*. *japonica*. There could be several reasons for the higher LMR in *C*. *japonica*. Since plants grown under high soil resource availability allocate more biomass to foliage at the expense of roots [68, and references therein]. *C*. *japonica*, which is often planted in fertile wet sites, might allocate more biomass to foliage than *C*. *obtusa*, which is planted in less fertile and dry sites. Another possible reason is related to the shoot and crown form of *C*. *japonica*. Generally, the leaf area index (≈leaf biomass) is larger in canopies where the light absorption coefficient (*k*) of Lambert–Beer law is smaller because light penetrates deeper into the canopy [55]. One of the factors that decreases *k* is a steep foliage inclination [56, and referenes therein], and *C*. *japonica*, which has three-dimensional shoots, had a lower *k* (0.38) than *C*. *obtusa* with planar shoots (0.97, but only one data entry was available, Table 1). Therefore, the shoot morphology that allows light penetration deeper into the crown may cause *C*. *japonica* to have a thick canopy (= high LMR).

### 3.2 Differences in traits related to water use

#### 3.2.1 Stomatal conductance and transpiration rate

**Fig 5:**
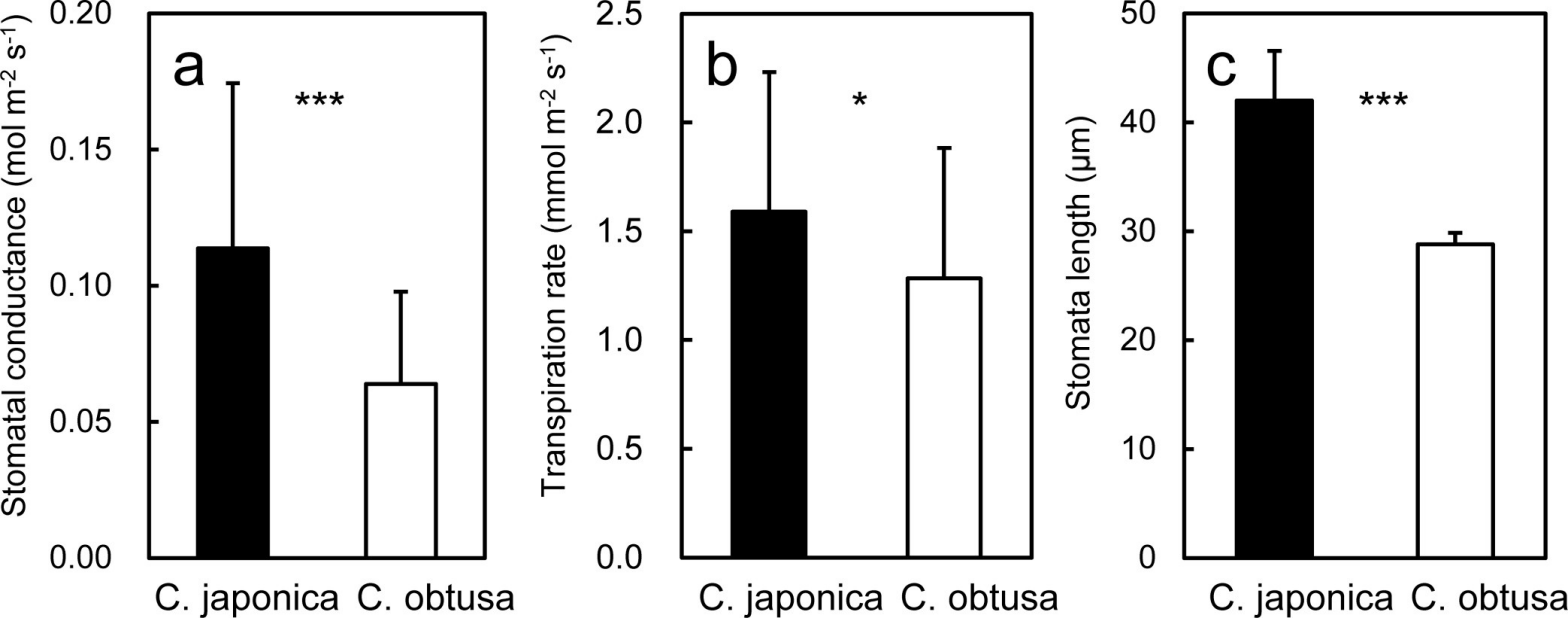
Leaf traits related to water relation of *C*. *japonica* and *C*. *obtusa*. In *C*. *japonica*, each trait is presented on a shoot silhouette area basis and projected needle area basis. Differences between species were determined by a t-test; ***, P<0.001; *, P<0.05.

*C*. *japonica* exhibited 1.7- and 1.5-fold higher stomatal conductance to CO_2_ (gs_an_) and transpiration rate (E_an_), respectively, compared with *C*. *obtusa* (Fig 5A and 5B). In concert with this, stomatal distribution and anatomy differed largely between the species. Coniferous stomata are generally distributed unevenly on foliar surfaces, forming a species-specific pattern of stomatal clusters called a “white band”. *C*. *japonica* has two thick white bands on every surface of the triangular pyramid-shaped needles, whereas *C*. *obtusa* has y-shaped white bands along the rims only on the abaxial surface of its scales [70–72]. In addition to this larger proportional area of white bands to the total foliage surface, the stomatal diameter of *C*. *japonica* was 1.5 times higher than that of *C*. *obtusa* (Fig 5C).

Despite the lower gs_an_ and E_an_ in *C*. *obtusa*, Amax_an_ was not significantly different between the species (Fig 2A), suggesting that water use efficiency, that is, gs_an_ or E_an_ divided by Amax_an_, is higher in *C*. *obtusa* than in *C*. *japonica*. The lower gs_an_ and E_an_ and higher water use efficiency are regarded as adaptations of drought [73], which supports the hypothesis that *C*. *obtusa* is more drought tolerant than *C*. *japonica*. However, differences in drought resistance is represented not only by maximum gas exchange rates but also by the magnitude and speed of the stomatal response to changing environments [74, 75]. Although stomatal sensitivity are less often measured than potential gas exchange rates and they are not collected in SugiHinokiDB, some studies that estimated canopy stomatal conductance by sap flow measurements suggested that mature *C*. *japonica* trees were less sensitive to increasing VPD than *C*. *obtusa* [76, 77]. Similar less sensitivity of stomatal response of mature *C*. *japonica* trees has recently been reported under artificial soil drying experiments [78].

#### 3.2.2 Foliage PV curve parameters

**Fig 6:**
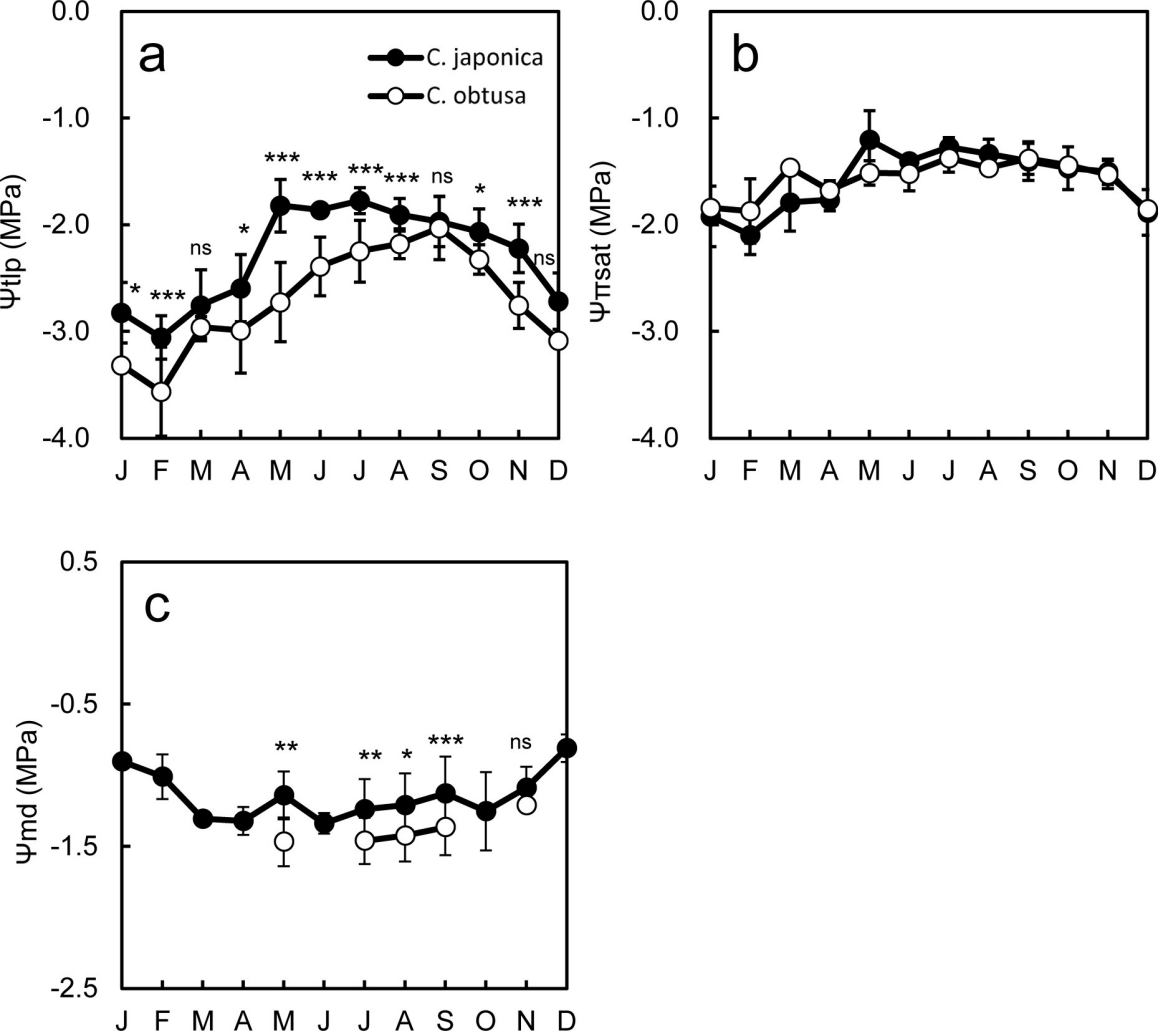
Seasonal changes in pressure volume parameters and midday leaf water potential. The effects of the independent variables (species, sampling month, and their interaction as fixed effects) on the dependent variables (each pressure-volume parameter) were evaluated using a linear mixed model [69]. Type III tests were performed for fixed effects (Wald-type test). Significant interspecific differences were on Ψtlp and Ψmd (*P* < 0.0001, Type III test, Table 2). Effect of the month was also significant for all tested pressure-volume parameters (*P* < 0.0001, Type III test, Table 2). Asterisk in the figure indicates significant differences between species in each month (ANOVA, *, *P* < 0.05; **, *P* < 0.01; ***, *P* < 0.001; ns, not significant). The bars indicate standard division.

Among the parameters of the pressure-volume curve, Ψ_tlp_ (foliage water potential at the turgor loss point) showed the clearest species difference: Ψ_tlp_ was larger in *C*. *obtusa* than in *C*. *japonica* throughout the year (Fig 6A, Table 2). Since plants with more negative Ψ_tlp_ are able to maintain cell turgor pressure under drought stress, thereby sustaining stomatal conductance, photosynthesis and growth, Ψ_tlp_ is thought to be predictive of the drought tolerance of plant species [79–84]. Indeed, midday foliage water potential (Ψ_md_) was significantly lower in *C*. *obtusa* than in *C*. *japonica* during summer (Jul to Sep, Fig 6C, Table 2) indicating that *C*. *obtusa* continued to open stomata until the water potential more decreased than that of *C*. *japonica*. This supported the hypothesis that *C*. *obtusa* is more drought tolerant than *C*. *japonica*. There are three possible ways in which Ψtlp becomes more negative: the accumulation of solutes such as sugar (decreases Ψπsat), a reduction in the symplastic water content through the redistribution of more water outside the cell walls (decreases Ψπsat) and an increase in cell wall flexibility (decreases ℇ) [15]. Ψπsat was similar between *C*. *obtusa* and *C*. *japonica* throughout the year (Fig 6B, Table 2). Unfortunately, ℇ-values were limited in the database, and we could not make reliable comparisons of monthly ℇ between the species. Nevertheless, the small difference in Ψπsat suggests that the lower Ψtlp of *C*. *obtusa* might be due to its presumed smaller ℇ. Species differences in Ψtlp are more often correlated with Ψπsat than with ℇ since a unit decrease in Ψπsat causes a larger decrease in Ψtlp than in ℇ [15, 75, 85, 86]. However, as was shown in the present study, there are also studies that showed a correlation between Ψtlp and ℇ [87].

**Table 2:** Results of type III test for seasonal changes in pressure volume parameters and midday leaf water potential.

	Ψtlp			Ψπsat			Ψmid		
	*d*.*f*.	*F*	*P*-value	*d*.*f*.	*F*	*P*-value	*d*.*f*.	*F*	*P*-value
Species	288	88.8	<0.0001	221	0.1	ns	282	19.4	<0.0001
Month	288	51.4	<0.0001	221	15.4	<0.0001	282	11.9	<0.0001
Species × Month	288	4.9	<0.0001	221	2.4	<0.01	282	0.3	ns

ns means not significant (*P*>0.05).

**Fig 7:**
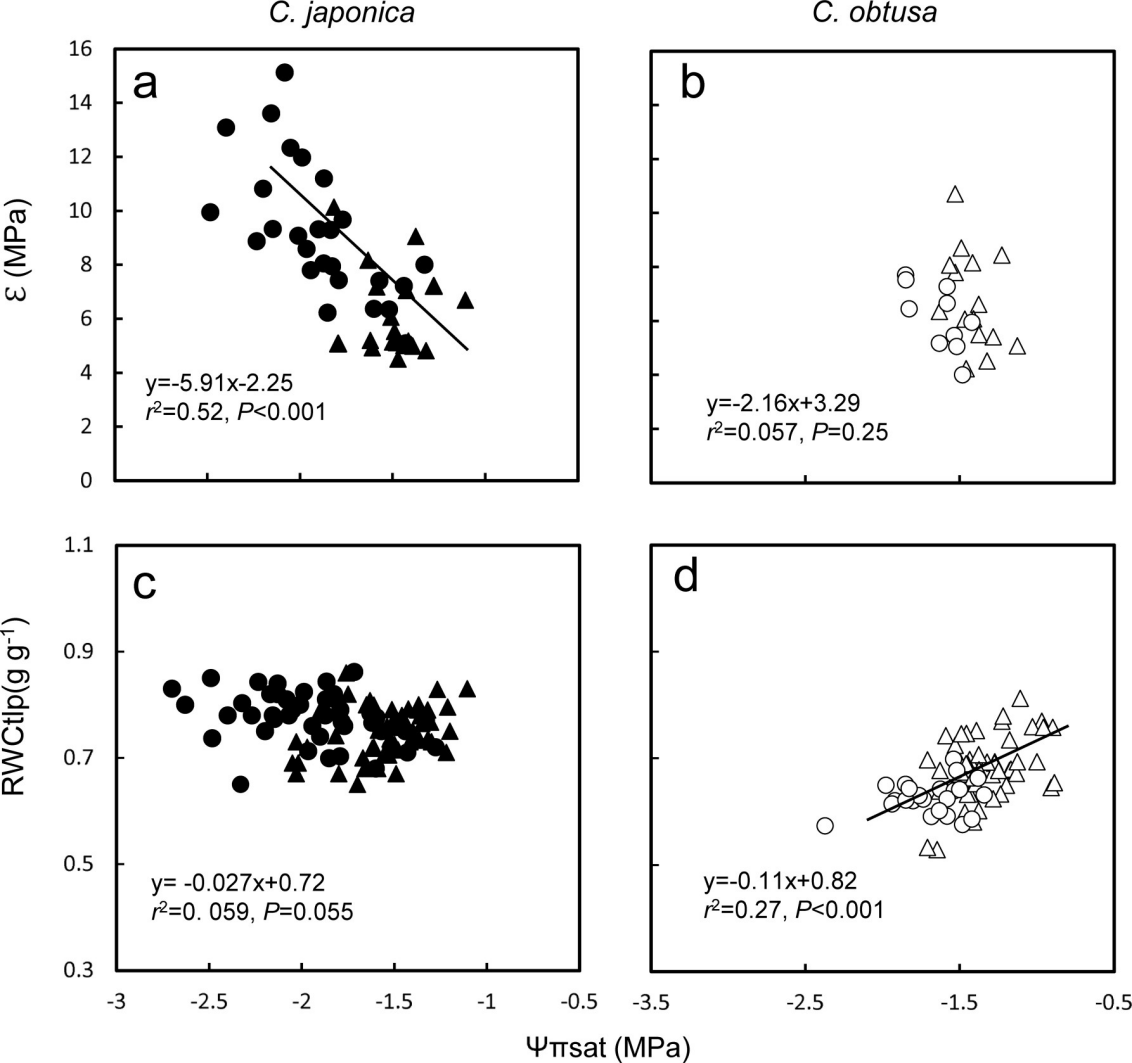
Relationship between pressure-volume parameters. Data measured from May-Oct and Nov-Apr were pooled. Filled triangle, *C*. *japonica* in May-Oct; Filled circle, *C*. *japonica* in Nov-Apr; Open triangle, *C*. *obtusa* in May-Oct; Open circle, *C*. *obtusa* in Nov-Apr. Regression lines are shown where they are significant (*P* < 0.05).

The other marked difference in the PV curve parameters was that *C*. *japonica* had higher ℇ in winter (Nov-Apr) compared to summer (May-Oct), whereas *C*. *obtusa* showed no seasonal changes in ℇ (Fig 7A and 7B). Winter increases in ℇ were also reported for *Eucalyptus* species [88], Taiwan cedar [89] and Patagonian woody shrubs [90, 91], which are thought to reduce physical injury to cell membranes by making cell walls more rigid (higher ℇ) during extracellular freezing and/or thawing processes. However, if increases in ℇ occur in response to freezing resistance, it is not clear why only *C*. *japonica* exhibited this response while both species exhibited a winter decrease in Ψπsat (Fig 7A and 7B), which is also a well-known response to freezing [92, 93]. Considering that *C*. *japonica* is more water demanding, another explanation may be possible for the winter increase in ℇ—the ‘cell water conservation hypothesis’ [15, 94]. Theoretically, reductions in Ψπsat decrease both Ψtlp and RWCtlp. However, a coordinated reduction in Ψπsat and an increase in ℇ would lower Ψtlp while maintaining a constant RWCtlp, which would result in tolerance for freezing and also prevent dangerous cell dehydration and shrinkage. In *C*. *japonica*, ℇ was negatively correlated with Ψπsat (*P* < 0.001, Fig 7A), and in accordance with the theory, RWCtlp remained constant irrespective of Ψπsat (Fig 7C). In contrast, in *C*. *obtusa*, where reductions in Ψπsat did not accompany increases in ℇ (*P* > 0.05, Fig 7B), RWCtlp decreased with reductions in Ψπsat (*P* < 0.001, Fig 7D). Plant species have a minimum cellular water content to maintain metabolic functions [95]. Since *C*. *japonica* has a higher minimum tissue water requirement for survival than *C*. *obtusa* [96], it may adjust ℇ in coordination with Ψπsat to constantly maintain RWCtlp above this minimum tissue water content.

#### 3.2.3 Hydraulic architecture

**Fig 8:**
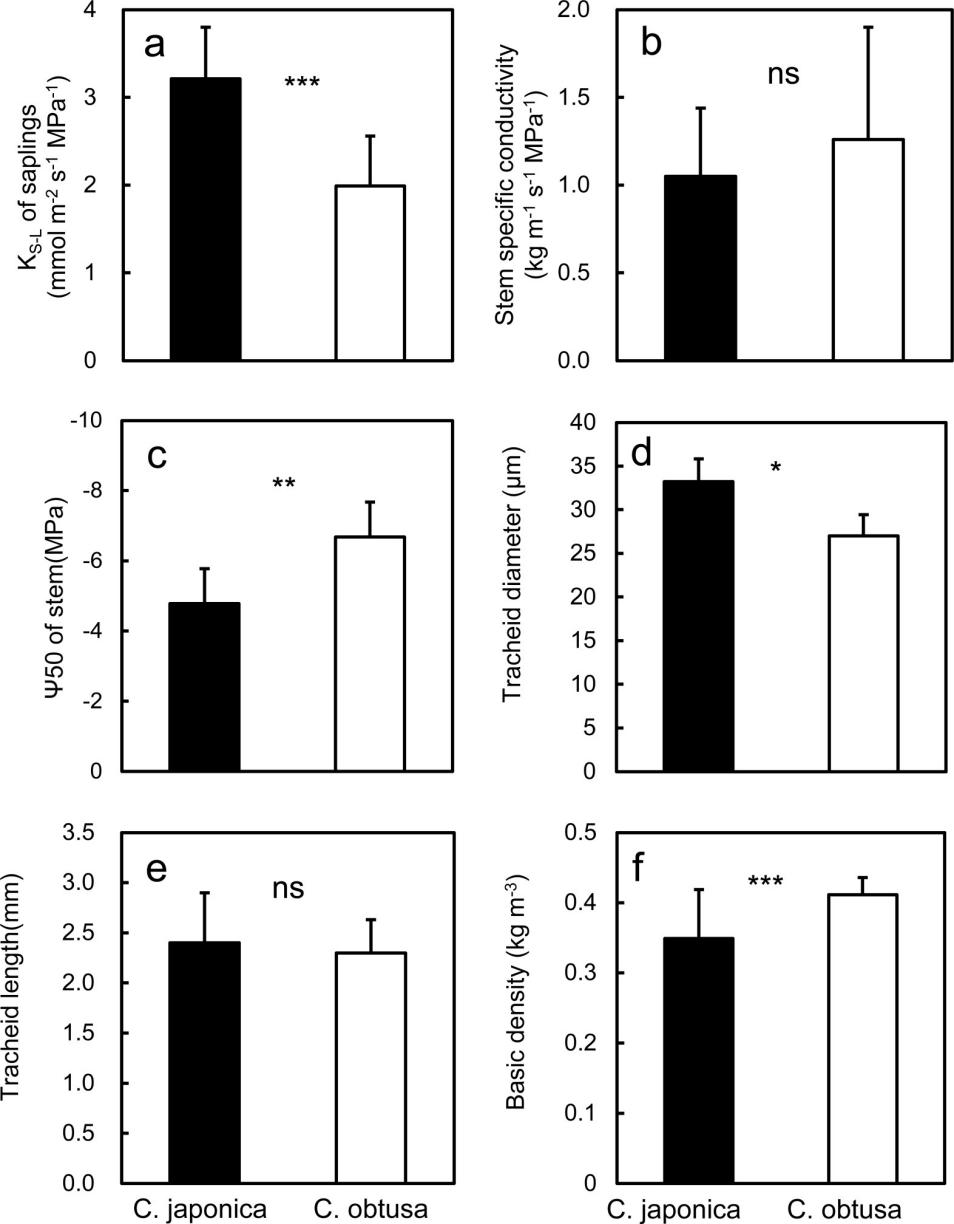
Stem hydraulics and xylem anatomy. Differences between the species were examined by *t*-test; ***, *P* < 0.001; **, *P* < 0.01; *, *P* < 0.05; ns, not significant.

Soil-to-foliage hydraulic conductance (K_S-L_), an index of whole-plant hydraulic efficiency, was 1.6 times higher in seedlings of *C*. *japonica* than in seedlings of *C*. *obtusa* (Fig 8A). Hydraulic conductance is a major determinant of plant water status and stomatal behaviour [97, 98] because of the relationship E_an_ = K_S-L_ (Ψ_pd_-Ψ_L_), where Ψ_pd_ is the foliage water potential measured at predawn, which represents the soil water potential, and Ψ_L_ is the foliage water potential. In accordance with this relationship, *C*. *japonica*, with a higher K_S-L_, had a higher transpiration rate than *C*. *obtusa* (Fig 5B), maintaining a higher midday foliage water potential (Fig 6C). Extensive measurements of K_S-L_ across species have revealed the adaptive significance of hydraulic conductance across functional groups; that is, pioneer, mesic and drought-avoiding species have higher hydraulic conductance than late successional, xeric and drought-tolerant species [98–102] The higher K_S-L_ in *C*. *japonica* than in *C*. *obtusa* appears in line with this. However, since K_S-L_ depends on the water transport distance, here, we used only seedling data where comparisons at similar sizes (ca. 1 m in height) were possible and are not sure whether this is also true for adult trees.

Since K_S-L_ is determined by the conductivity of all organs along the whole water transport pathway, knowledge on the hydraulic conductivity of each organ would provide more insight for water use. Unfortunately, we still have limited knowledge on organ hydraulic conductivity of these species. The only available information, i.e., stem-specific conductivity presented as hydraulic conductance per stem sapwood area (K_stem_), was not significantly different between the two species (Fig 8B), suggesting that the higher K_S-L_ of *C*. *japonica* could be due to the higher foliage and/or root hydraulic conductivity of this species.

In addition to hydraulic efficiency, hydraulic safety is another important factor of hydraulic architecture [103]. Although we have even less information for hydraulic safety than for hydraulic conductivity, *C*. *obtusa* is more resistant to embolism than *C*. *japonica* (presented by 1.4 times lower Ψ50, Fig 8C) [104, 105]. This means that *C*. *obtusa* can endure more severe negative pressure than *C*. *japonica* [17], which supports the empirical knowledge that this species is more tolerant to drought. However, in Japan, where precipitation is generally high throughout the year, plants rarely experience such extreme negative pressure, represented by the Ψ50 (-6.7 MPa). Therefore, the extent to which the differences in Ψ50 are relevant to their habitat preference is not obvious. Recently, Ψe, which is the xylem pressure at the start of conductivity loss, has been considered to be a more suitable index for drought tolerance than Ψ50 in nonextreme habitats [103]. Although Ψe is less focused on than Ψ50, studies show that plants control stomatal conductance to maintain xylem pressure near Ψe [106–108], indicating that Ψe could be a key factor linking stomatal control and xylem pressure. Furthermore, in conifers, hydraulic vulnerability segmentation, that is, a lower resistance to embolism in the distal segments that ensures the safety of the more proximal stems, is also known a common hydraulic strategy [109]. If so, not the hydraulic resistance of stems themselves but the differences in resistance between distal segments and more proximal segments (e.g., differences in Ψ50 between leaves and stems) may better represent species’ hydraulic strategy. Measurements of these traits would lead to a better understanding of the drought response of these species.

The tracheid diameter of the stem xylem was 1.26 times larger in *C*. *japonica* than in *C*. *obtusa*, and the length was not significantly different (Fig 8D and 8E). The basic density of stem wood (wood density) was lower in *C*. *japonica* than in *C*. *obtusa* (Fig 8F). Generally, the tracheid structure is considered to be closely related to water transport efficiency and drought safety, such as cavitation in the xylem [110, 111]. Conducting efficiency increases with tracheid diameter according to the Hagen–Poiseuille law, and it is also correlated with the tracheid length because a longer tracheid can result in conductive pits in the end walls, where water flow is significantly limited, being farther apart [38, 112, 113]. If so, stem specific conductivity should be higher in *C*. *japonica* than in *C*. *obtusa*, but it was not significantly different between the species (Fig 8B). The reason is not clear, but the pit structure, which could affect conductivity and for which we have no information for these species, might play a role [110, 111]. A more negative Ψ50 is also known to be associated with a smaller tracheid diameter and greater basic density because it requires mechanical strength to support the xylem conduit against implosion caused by negative pressures [114–116]. The smaller tracheid diameter, higher wood density and more negative xylem Ψ50 in *C*. *obtusa* compared with *C*. *japonica* are in line with our hypothesis.

### 3.3 Age and height dependency

Tree age and/or size, especially height, usually affect many foliar functional traits, such as photosynthetic traits, stomatal behaviour, morphology, water use and nutrient concentrations, in various tree species, including angiosperms and gymnosperms in tropical, temperate, boreal and even semiarid areas [21, 117–127]. These age- and/or size-related foliage changes are important for understanding the forest growth rate, timber yield and carbon balance [124] and also contribute to accurate impact assessment of future climate change on forests. In this section, we demonstrate the effects of tree age and/or height on foliar nutrient concentrations (N, P, K), SLA and Ψ_md_ using the large datasets obtained from SugiHinokiDB.

#### 3.3.1 Foliage nutrient contents

**Fig 9:**
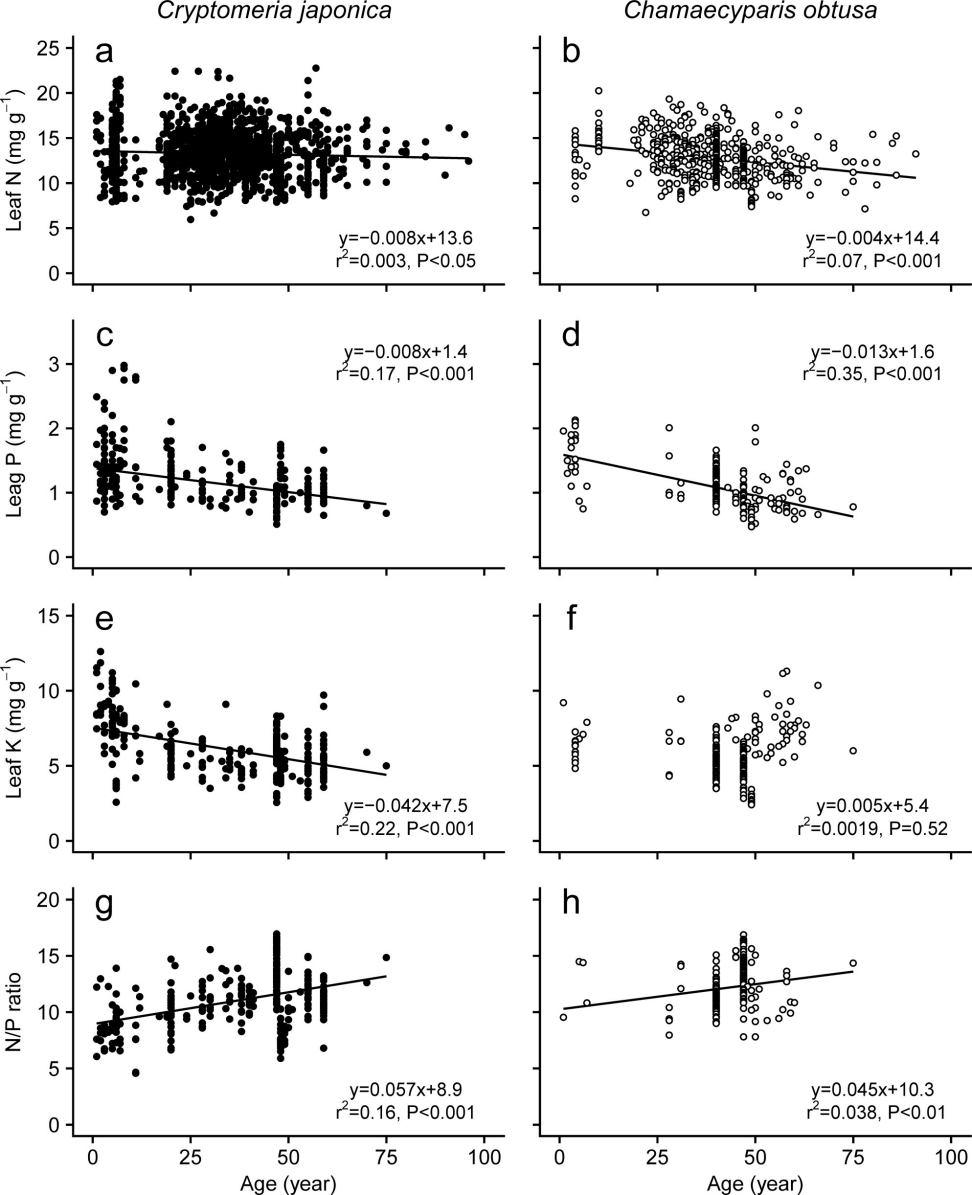
Changes in leaf nutrient contents with tree age. Data from plants grown under experimental conditions or in pots were excluded. Data from plants younger than 100 years old are shown. Regression lines are shown where they are significant (*P* < 0.05).

**Fig 10:**
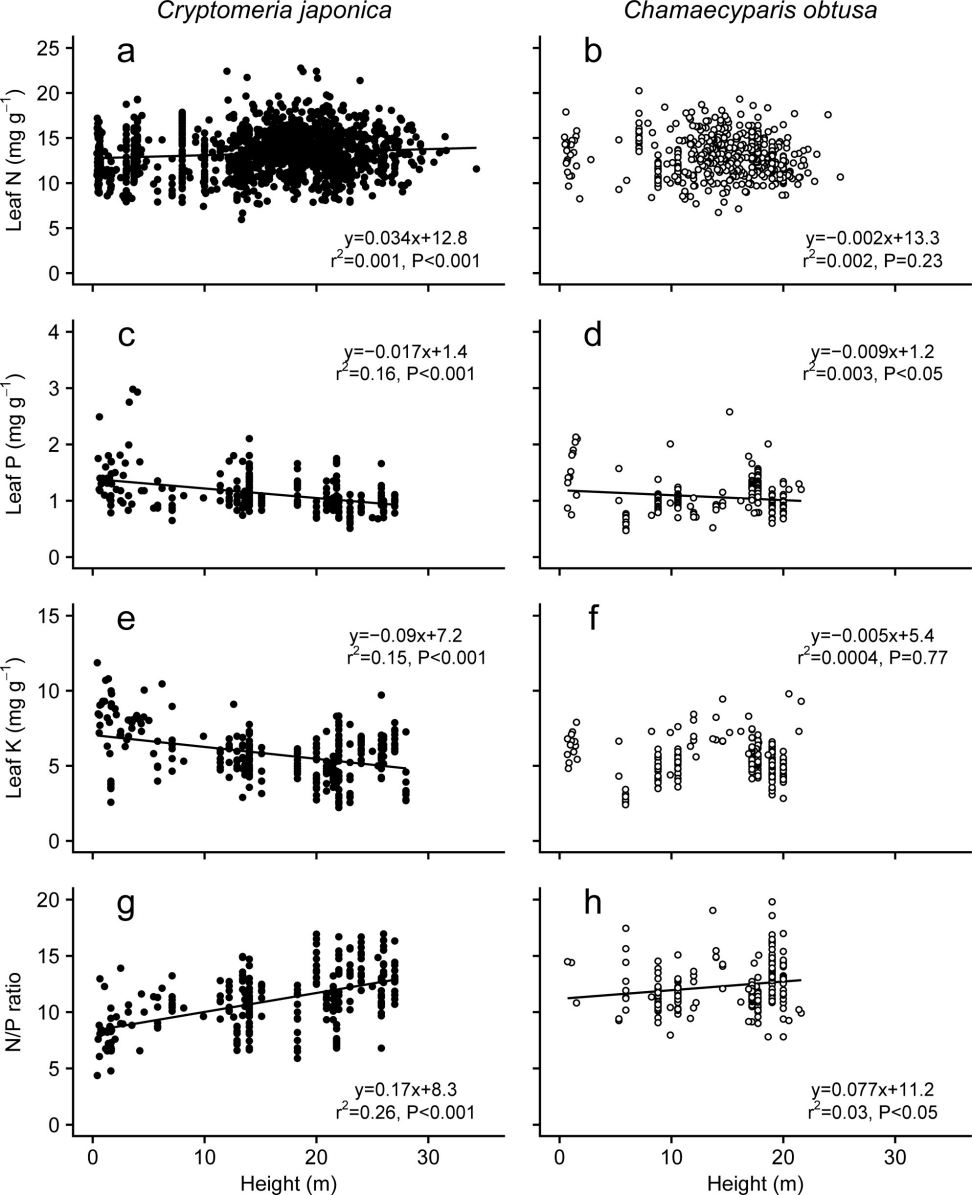
Changes in leaf nutrient contents with tree height. Data from plants grown under experimental conditions or in pots were excluded. Regression lines are shown where they are significant (*P* < 0.05).

Changes in foliar nutrient concentrations with tree age and/or height showed different patterns depending on tree species and nutrient type (Figs 9 and 10). The foliar N concentration decreased with the age classes of *C*. *japonica* and *C*. *obtusa* (Fig 9A and 9B), whereas it was slightly increased with height in *C*. *japonica* and unrelated to height in *C*. *obtusa* (Fig 10A and 10B). Foliar P and K concentrations also decreased significantly with age class and height in *C*. *japonica* (Figs 9C, 9E, 10C and 10E). A similar age- and height-dependent reduction in the P concentration was observed in *C*. *obtusa* (Figs 9D and 10B), although the K concentration was constant with age and height (Figs 9F and 10F). Previous studies also showed various patterns of age /size dependency of nutrient contents. Several cases indicate an increase in the foliar nutrient content with tree size and age [128,], while many other cases showed decreased [19, 20, 23, 24, 25, 129] or constant [24, 122, 123, 128, 130, 131] foliar nutrient. One of the possible causes of these inconsistency might be sampling effects caused by rather small datasets [132]. However, the influence of sampling effects seems to be small in the present study because of the large data sets used to examine the relationships (200–1500 data points, forest age up to 80 years old, tree height up to 40 m). Increasing nutrient accumulation to living biomass and coarse woody debris with forest development may cause decreased foliar nutrient concentrations with tree age and height through a reduction in soil nutrient availability [21, 128, 130]. Indeed, a reduction in soil nutrient concentrations with forest development has been reported in the early stages of forest growth [133–135]. In addition, higher drought stress, such as hydraulic limitation with tree height, promotes the investment of carbon into foliage to protect against dehydration, which results in dilution of the foliar nutrient [136–138]. These could be the causes of the ontogenetic reduction of foliar nutrient. In contrast, several authors have suggested that tree size- or age-related foliar N contents may have a unimodal relationship rather than a simple linear relationship [137–139]. If nonlinear changes in foliar N occurred in each *C*. *japonica* and *C*. *obtusa* stand, it may be difficult to identify a clear linear relationship of N concentration with tree size and height by using the present pooled analysis.

The reason that *C*. *japonica* and *C*. *obtusa* showed different age/height dependency is not clear. However, they are different in growth rate, nutrient demand and growth habitats. It is known that *C*. *japonica* forests, which are planted under moister conditions in nutrient-rich soil, are faster in nutrient dynamics than *C*. *obtusa* forests, which usually grow on upper slopes with poor nutrient and water availability [140, 141]. These differences could affect the age- and height-dependent changes in foliar nutrients.

The stoichiometry of N to P significantly increased with tree age and height (Figs 9G, 9H, 10G and 10H). The NP ratio is an index of soil nutrient limitation, i. e., P limitation occurs if the ratio is higher than 16, N limitation typically occurs if the ratio is lower than 14, and N and P are co-limiting if the value is 14–16 [142]. The maximum NP ratio of *C*. *japonica* is 16.9, and 87.2% of all data points have values of 14 or lower, suggesting that *C*. *japonica* stands are generally N-limited. However, since the NP ratio increases with age, the stands shift from being N- to being relatively P-limited with maturity. *C*. *obtusa* also has an NP ratio of 14 or less, and are considered to be in the N-limited range (Fig 9H).

#### 3.3.2 Foliage water potential and specific leaf area (SLA)

**Fig 11:**
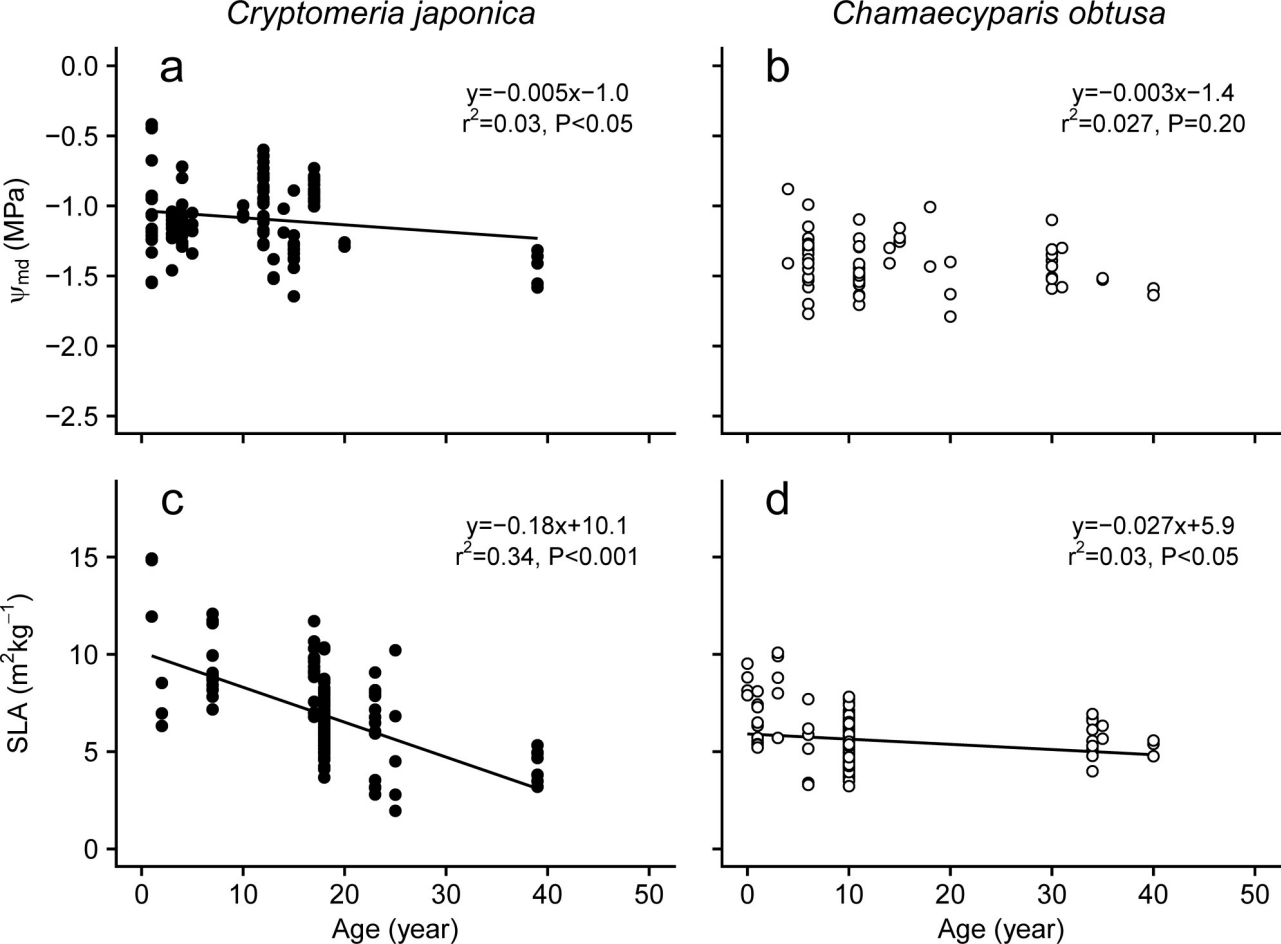
Changes in midday leaf water potential (Ψmd) and SLA with tree age. Data from plants grown under experimental conditions or in pots were excluded. Data from plants younger than 100 years old are shown. Regression lines are shown where they are significant (*P* < 0.05).

**Fig 12:**
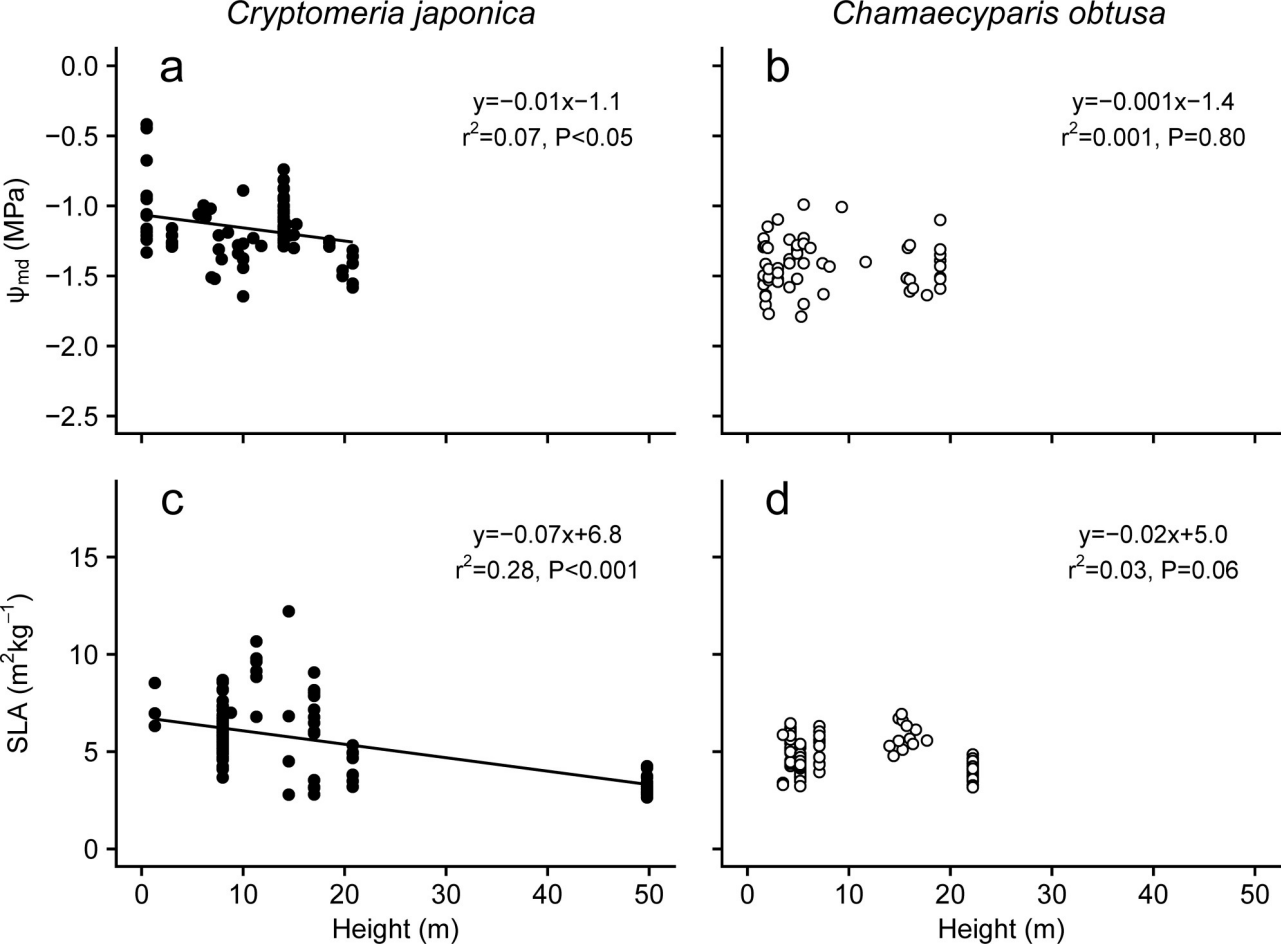
Changes in midday foliage water potential (Ψmd) and SLA with tree height. Data from plants grown under experimental conditions or in pots were excluded. Regression lines are shown where they are significant (*P* < 0.05).

As tree height increases, drought stress increases in the upper part of the canopy, which in turn affects morphological and physiological responses of tree foliage [120, 124]. The foliar midday water potential, Ψmd, which indicates the degree of tree drought stress, significantly decreased with height and age in *C*. *japonica* trees (Figs 11A and 12A), but not in *C*. *obtusa* trees (Figs 11B and 12B). The reduction in Ψmd with height have been reported for various tree species worldwide and cause hydraulic limitations, such as a reduction in photosynthesis through stomatal limitations [118, 139, 143–146]. Interestingly, the recovery of photosynthetic ability by grafting canopy tree shoots onto saplings of *C*. *japonica* supported the occurrence of hydraulic limitations in tall trees of this species [147]. The slopes of Ψmd with the tree height are −0.0103 and −0.0089 Mpa m^−1^ in *C*. *japonica* and *C*. *obtusa*, respectively. These slopes are smaller than those of tropical rainforest trees (−0.0282 Mpa m^−1^, Kenzo et al., 2015) and in the range of the values of temperate conifers *Sequoia sempervirens* (−0.0100–−0.0113 Mpa m^-1^) and Douglas fir (−0.019 Mpa m^-1^) [120, 139]. In general, to tolerate a decrease in Ψ_md_, the foliar water potential at the turgor loss point (Ψ_tlp_) must be reduced [84, 148]. Foliar osmotic adjustment as well as structural strength to withstand low negative pressure to achieve lower Ψ_tlp_ and foliage strength is accompanied by a decrease in SLA. In fact, the SLA of *C*. *japonica*, whose Ψ_md_ significantly decreased with tree age and height, decreased with tree height and age (Figs 11C and 12D). Many studies have reported that SLA decreases with tree age and height [120–123, 125, 132, 149, 150]. On the other hand, the SLA of *C*. *obtusa* did not show a significant change with tree height, though it slightly decreased with tree age (Figs 11D and 12D). Although the reason is not clear, the smaller changes in SLA of *C*. *obtusa* might be related to its higher resistance to drought stress. It is also possible that the smaller size range in *C*. *obtusa* compared with *C*. *japonica* may cause this constant SLA. [151] recently reported that SLA decreased significantly at a single canopy height in a *C*. *obtusa* tree. Thus, further data collection on older and taller *C*. *obtusa* trees is needed to understand tree size dependency with respect to SLA.

### 3.4 Comparison with broader species in *Cupressaceae*

The Cupressaceae family, to which *C*. *japonica* and *C*. *obtusa* belong, consists of more than 100 species with a marked diversity in physiology, morphology and habitat preference [152]. How much do the contrasts we found between *C*. *japonica* and *C*. *obtusa* in the present study account for the ranges in traits exhibited by Cupressaceae species? Here, we compare traits related to drought tolerance between the two species and other Cupressaceae species using data from [152] to gain more insight into the ecological characteristics of these species.

**Fig 13:**
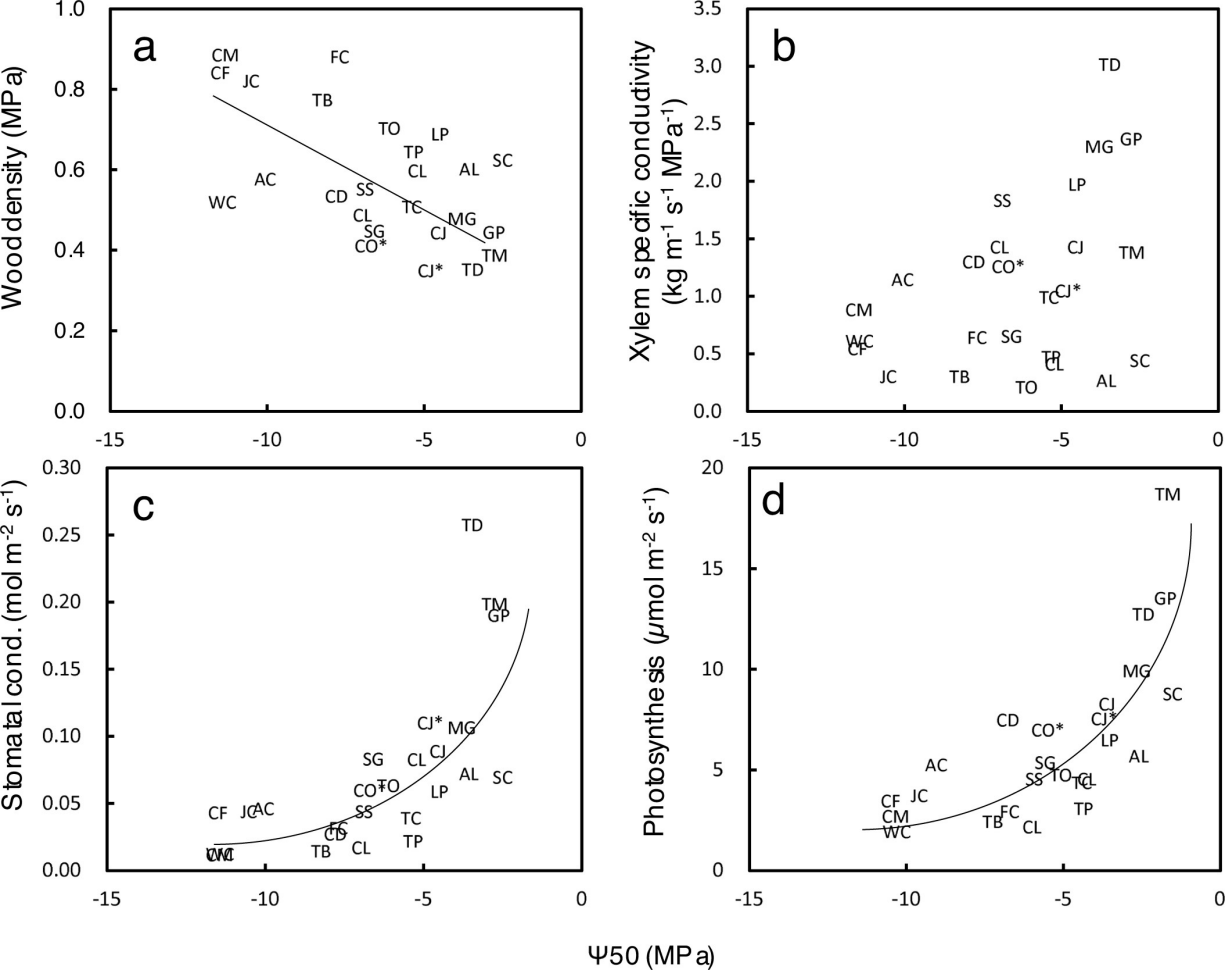
Relationship between leaf and stem hydraulic properties in Cupressaceae species. Abbreviations of species names are shown in Table 3. Species in bold type with an asterisk are from this study. Regression lines are shown where they are significant (*P* < 0.05).

Cupressaceae species first appeared in the warm and humid Mesozoic and differentiated in the cool and dry Cenozoic [153]. Reflecting the climatic conditions under which each species evolves, early diverging species prefer mesic-hydric habitats, while derived species are adapted to arid climates [152]. As a result of these adaptations, the species exhibit a gradient of foliage morphology (e.g. from needle-like foliage in basal species to small scaly foliage in more derived species), drought tolerance and water use characteristics [38, 107, 154]. Fig 13 shows such gradients across Cuperessaceae. Basal species (e.g., *Glyptostrobus*, *Taxodium*, *Metasequoia*) that grow in moist environments had lower Ψ_50_ with higher wood density (Fig 13A), lower xylem specific conductivity (Fig 13B), low stomatal conductance (Fig 13C), and a lower photosynthetic rate (Fig 13D). On the opposite end of the axis are derived species growing in dry environments such as *Callitris*, *Juniperus*, and *Widdringtonia* (Fig 13A–13D).

**Table 3:** Species name and abbreviations used in Fig 13.

Species	Abbr.	Phenology, native habitat
*Athrotaxis laxifolia*	AL	E, Montane forests, Tasmania
*Austrocedrus chilensis*	AC	E, The Andes, Chile and Argentina
*Callitris macleayana*	CM	E, Mesic-dry forests, eastern Australia
*Calocedrus decurrens*	CD	E, Montane forests, United States–Mexican Pacific Coast
*Chamaecyparis lawsoniana*	CL	E, Mixed forests, Oregon to northern California
*Chamaecyparis obtusa* (This study)	CO*	E, Mixed evergreen forests, Japan and Taiwan
*Cryptomeria japonica*	CJ	E, Mixed evergreen forests, Japan
*Cryptomeria japonica* (This study)	CJ*	E, Mixed evergreen forests, Japan
*Cunninghamia lanceolata*	CL	E, Mixed broad-leaved forests of southeast Asia
*Cupressus forbesii*	CF	E, Chaparral, southern California, northern Mexico
*Fitzroya cupressoides*	FC	E, Evergreen rainforest, Chile
*Glyptostrobus pensilis*	GP	D, Riparian, southern China
*Juniperus californica*	JC	E, Desert, southern California to northern Mexico
*Libocedrus plumosa*	LP	E, Mixed conifer rainforests, New Zealand
*Metasequoia glyptostroboides*	MG	D, Mesic mixed forests, central China
*Sciadopitys verticillata*	SC	E, Temperate moist forests, Japan
*Sequoia sempervirens*	SS	E, northern coastal California
*Sequoiadendron giganteum*	SG	E, Sierra Nevada, California
*Taiwania cryptomeroides*	TC	E, Cool temperate forests, Asia
*Taxodium distichum*	TD	D, Riparian regions in southeastern United States
*Taxodium mucronatum*	TM	D, Southern Texas, Mexico, Central America
*Taxus baccata*	TB	E, Broadly distributed across Europe
*Thuja plicata*	TP	E, Mixed coniferous forests, United States Pacific northwest
*Thujopsis dolabrata*	TO	E, Coastal and montane Japan
*Widdringtonia cedarbergensis*	WC	E, Fynbos, South Africa

Data of *Cryptomeria japonica* and *Chamaecyparis obtusa* are from this study, and other data are from Pittermann et al. 2012.

‘E’ represents for evergreen and ‘D’ represents for deciduous.

The traits of *C*. *japonica* and *C*. *obtusa* also fell on these correlation lines (Fig 13). Among these axes, *C*. *japonica*, which is more basal than *C*. *obtusa*, was located next to the lowest Ψ_50_ (= the highest gas exchange) group, consisting of species of *Glyptostrobus*, *Taxodium*, and *Metasequoia*. This seems reasonable given that these genera form the same clade as *C*. *japonica in Cryptomeria*. *C*. *obtusa*, which had a slightly lower Ψ_50_ than *C*. *japonica*, was located almost in the middle of the axes where close relatives, such as *Chamaecyparis lawsonia* (the same genus with *C*. *obtusa*) and *Thuja* and *Thujopsis* species, occurred. However, across all Cupressaceae species, the difference between *C*. *japonica* and *C*. *obtusa* was not large, nor was *C*. *obtusa* especially high in drought tolerance within the Cupressaceae species. Japan, which is surrounded by the sea and has high-altitude mountains, has not suffered an extremely dry climate, even after the Cenozoic era, and only a few species of *Juniperus* with strong drought tolerance grow in alpine and coastal areas. Rather, there exist many relict genera, such as *Chamaecyparis*, *Thuja* and *Thujopsis*, which are located in the middle of the axis. This suggests that the mild climate of the Japanese archipelago became a refugia of these species with moderate drought tolerance in the arid Cenozoic [155]. However, high drought tolerance is generally achieved at the expense of a low growth rate. Therefore, the moderate drought tolerance of *C*. *obtusa* ensures a moderate growth rate and makes the species a major alternative to *C*. *japonica* at relatively dry forestry sites in Japan.

### 3.5 Implication for climate change

The detailed comparison of two major species highlights the importance of incorporating trait data into forest ecosystem models for more accurately predicting responses to climate change. Parameterizing models with various trait data enables us to obtain more realistic responses of trees and predictions. Recent studies demonstrate the limitations of plant functional type approaches and claim the need for trait-based approaches, for example, hydraulic responses [156]. Most models at present are plant functional-type based; in other words, default parameters are prepared for different plant functional types; however, recent trait data compilation studies, such as this study, clearly demonstrate the diversity of traits among tree species even in the same plant functional type. The latest study even proposes flexible trait models for the next generation of vegetation models [157]. These trait-based approaches would succeed more easily in manmade pure forests than in mixed natural forests.

More specifically, our study provides important implications about possible differences in the species’ responses to climate change: no differences in SLA and photosynthetic ability per needle area were found between *C*. *japonica* and *C*. *obtusa*; on the other hand, the different structures of shoots and canopies between the two species may cause a difference in the amount of photosynthetic production per day. In particular, in the early planting period from just after plantation to canopy closure, *C*. *japonica m*ay have more advantages than *C*. *obtusa* due to its high photosynthetic production under bright light conditions. However, a big-leaf model, which is a typical photosynthesis model, does not explicitly describe the photosynthetic ability per part, structure or mass [48]. The response of stomata to clpimate change, such as the response of stomatal conductance to vapor pressure deficit (VPD), may differ between *C*. *japonica* and *C*. *obtusa*, but it is not clear at this stage due to a lack of data. In other words, it is possible to accurately grasp the type and amount of data that are lacking for modelling by using the database, which allows us to make an experimental plan to efficiently collect data according to a specific purpose. Regarding water use, we found various differences in related traits between *C*. *japonica* and *C*. *obtusa*; however, in general, most traits related to drought tolerance and xylem hydraulic safety except for gas exchange traits are rarely incorporated into models. This is perhaps partly because of the lack of data; drought tolerance and xylem hydraulic safety are new fields that have recently emerged.

In Japan, *C*. *obtusa* is a species planted in dry sites, while *C*. *japonica* is planted in wet sites; however, our compilation revealed that the drought tolerance of *C*. *obtusa* was moderate within the wide range of traits of the global Cupressaceae family. In fact, there are several reports of drought damage in *C*. *obtusa* forests, particularly in western Japan, which experiences more severe drought more often than in eastern Japan [158, 159]. These facts imply that it is very likely that not only *C*. *japonica* but also *C*. *obtusa* will suffer from possible more severe droughts induced by future climate change.

## 4. Conclusion

The present study challenged the empirical knowledge of two contrasting plantation species in Japan. The intensive analysis of plant trait database clearly supported traditional knowledge and empirical plantation management in *C*. *japonica* and *C*. *obtusa*, such as preferable planting sites for the two species (*C*. *japonica* on wet lower slopes and *C*. *obtusa* on dry ridges). Overall, *C*. *japonica* showed more pioneer-like properties. Although the photosynthesis per foliage area was similar to *C*. *obtusa*, *C*. *japonica* could be higher in photosynthetic production due to the high shoot-level light utilization efficiency. In addition, the high biomass allocation to the foliage and the low wood density of *C*. *japonica* result in a high stem volume yield. On the other hand, *C*. *obtusa* has high drought tolerance due to its lower transpiration rate, stomatal conductance and water potential at the foliar turgor loss point, whereas its photosynthesis at the shoot level is lower than that of *C*. *japonica*. Our finding that the most functional characteristics change according to tree age and/or height indicates that forest management also reflects a functional shift with the ontogeny of the tree. For example, to maximize tree production, fertilization to prevent foliar functional deterioration and thinning to compensate for the water supply can be considered with forest ageing. Within the global Cupressaceae family, both species have moderate drought tolerance and photosynthetic rates, and the traits may be consistent with the historical climate of the Japanese archipelago being warm and humid without severe drought. The relatively low tolerance of both species may indicate a weak ability to withstand severe dry events associated with future climate change. Although this database study provided a robust comparison between *C*. *japonica* and *C*. *obtusa*, there were several limitations. For example, data filtering and outlier handling will be necessary to obtain reliable results due to the inconsistent quality of the data and the variation in sample size. Additionally, the interaction between traits is often not considered in trait database studies because linking traits in a database studies due to the requirement of data filtering and its structure (traits usually handled in separate spreadsheets). Further studies will be needed as the interactions may provide a deeper understanding of interspecific differences in ecological traits.
